# A Novel Triazolopyridine-Based Spleen Tyrosine Kinase Inhibitor That Arrests Joint Inflammation

**DOI:** 10.1371/journal.pone.0145705

**Published:** 2016-01-12

**Authors:** Gregory D. Ferguson, Mercedes Delgado, Veronique Plantevin-Krenitsky, Kristen Jensen-Pergakes, R. J. Bates, Sanaa Torres, Maria Celeridad, Heather Brown, Kelven Burnett, Lisa Nadolny, Lida Tehrani, Garrick Packard, Barbra Pagarigan, Jason Haelewyn, Trish Nguyen, Li Xu, Yang Tang, Matthew Hickman, Frans Baculi, Steven Pierce, Keiji Miyazawa, Pilgrim Jackson, Philip Chamberlain, Laurie LeBrun, Weilin Xie, Brydon Bennett, Kate Blease

**Affiliations:** 1 Department of Inflammation Research, Celgene Corporation, San Diego, California, United States of America; 2 Department of Chemistry, Celgene Corporation, San Diego, California, United States of America; 3 Department of Pharmacology, Celgene Corporation, San Diego, California, United States of America; 4 Department of Biochemistry, Celgene Corporation, San Diego, California, United States of America; 5 Department of Tumor Cell Biology, Pfizer Corporation, San Diego, California, United States of America; 6 Department of Corporate Planning and Strategy, Kissei Pharmaceutical Company, Matsumoto City, Nagano, Japan; University of Torino, ITALY

## Abstract

Autoantibodies and the immunoreceptors to which they bind can contribute to the pathogenesis of autoimmune diseases such as rheumatoid arthritis (RA). Spleen Tyrosine Kinase (Syk) is a non-receptor tyrosine kinase with a central role in immunoreceptor (FcR) signaling and immune cell functionality. Syk kinase inhibitors have activity in antibody-dependent immune cell activation assays, in preclinical models of arthritis, and have progressed into clinical trials for RA and other autoimmune diseases. Here we describe the characterization of a novel triazolopyridine-based Syk kinase inhibitor, CC-509. This compound is a potent inhibitor of purified Syk enzyme, FcR-dependent and FcR-independent signaling in primary immune cells, and basophil activation in human whole blood. CC-509 is moderately selective across the kinome and against other non-kinase enzymes or receptors. Importantly, CC-509 was optimized away from and has modest activity against cellular KDR and Jak2, kinases that when inhibited in a preclinical and clinical setting may promote hypertension and neutropenia, respectively. In addition, CC-509 is orally bioavailable and displays dose-dependent efficacy in two rodent models of immune-inflammatory disease. In passive cutaneous anaphylaxis (PCA), CC-509 significantly inhibited skin edema. Moreover, CC-509 significantly reduced paw swelling and the tissue levels of pro-inflammatory cytokines RANTES and MIP-1α in the collagen-induced arthritis (CIA) model. In summary, CC-509 is a potent, moderately selective, and efficacious inhibitor of Syk that has a differentiated profile when compared to other Syk compounds that have progressed into the clinic for RA.

## Introduction

Autoimmune diseases are characterized by inappropriate immune responses that are mediated, in many cases, by pathogenic autoantibodies and the immunoreceptors (FcR) to which they bind. In rheumatoid arthritis (RA), for example, autoantibodies that recognize rheumatoid factor immunoglobulin or citrullinated proteins are established in disease etiology in some patients and are the basis of point-of-care diagnostic tests [1,2]. Moreover, RA susceptibility has been linked to distinct FcR haplotypes in certain populations [3,4]. Most currently approved RA therapies involve general immunosuppression or blockade of the proinflammatory molecules that are downstream of autoantibody action. It has been postulated that therapeutic efficacy in RA may also be achieved by blocking the production of or responsiveness to pathogenic autoantibodies [5,6].

Spleen tyrosine kinase (Syk) is a non-receptor tyrosine kinase expressed broadly in the hematopoietic lineage and an essential component in leukocyte signal transduction [7]. Syk binds to and is activated by immunoreceptors Fc-epsilon (FcεR), Fc-gamma (FcγR), or the B-cell receptor (BCR) in the appropriate cellular context. Although Syk (-/-) mice die shortly after birth, immune cells derived from these mice respond abnormally during antibody-dependent stimulation through FcεR or FcγR while B-cell differentiation and BCR functionality are similarly altered [8–11].Consistent with these deficits at the cellular level, mice with a conditional deletion of Syk are protected in antibody-mediated models of arthritis [12–14]. In addition, the levels, activation state, or recruitment status of Syk can also be increased or altered in certain human autoimmune diseases. Syk therefore has a central role in antibody-dependent immune cell activation and may mediate, at least in part, the pathophysiological mechanisms that underlie numerous instances of autoimmune disease.

Syk kinase inhibitors have emerged as promising therapeutic agents for the treatment of autoimmune diseases such as RA. Syk kinase inhibitors effectively block *in vitro* immune cell activation through the Fc-receptors and exhibit efficacy in rodent models of arthritis equivalent to that observed in Syk (-/-) mice, indicating that pharmacologic inhibition of Syk can promote near maximal levels of immune modulation [15–17]. A number of Syk inhibitors, most notably fostamatinib (R406/R788, Rigel Pharmaceuticals), have progressed into clinical trials [18]. As one of the first targeted small molecule therapeutics to be developed for RA, fostamatinib was innovative and provided valuable benchmarks for follow-on drug discovery and development efforts. However, insufficient late stage clinical efficacy and persistent tolerability issues led to the termination of fostamatinib clinical development in RA [19]. Another Syk inhibitor, BIIB057 (Biogen), was recently withdrawn prior to initiation of an RA Phase II trial [20]. Therefore, additional novel and differentiated Syk inhibitors will be required to establish Syk as a clinically validated target in RA.

Here we describe the identification of a potent, moderately selective, and orally bioavailable small molecule Syk kinase inhibitor based on a novel triazolopyridine core. The compound, CC-509, is a reversible, mixed ATP-competitive inhibitor of Syk that blocks FcR-dependent and FcR-independent cellular signaling, has favorable pharmacokinetic properties, and displays efficacy in two models of inflammation and arthritis. In addition, CC-509 has distinct cellular effects when run head-to-head against R406 and reduced activity against the biochemical targets thought to contribute to the side-effect profile observed in fostamatinib RA trials (i.e. KDR and Jak2). Taken together, our data indicate that CC-509 is clearly differentiated from R406 and suggests it may have a unique *in vivo* efficacy and safety profile when compared to other Syk kinase inhibitors in RA.

## Materials and Methods

### Compound

CC-509 (Celgene Corporation) was synthesized using standard chemical transformations and was fully characterized by NMR and mass spectrometry, as described in U.S. Patent 8299056-B2.

### Human blood, cell lines, and DNA constructs

Human whole blood was obtained, with donor consent, from the Scripps Normal Blood Donor Service (San Diego, CA). Human buffy coat blood fractions were obtained, with donor consent, from the San Diego Blood Bank (San Diego, CA). Mobilized CD14+ monocytes were purchased from and grown according to protocols provided by supplier (Lonza, Basel, CH). All cell lines used in this manuscript were either purchased from a commercial source (supplier indicated) or was obtained based on previous publication (reference cited). HEK293 T-REx™, Ramos NFAT β-lactamase, HEL Irf1-β-lactamase cells (Life Technologies, Grand Island, NY), murine BaF/3 cells (ATCC, Manassas, VA), HEK293 expressing human KDR (Sibtech, Brookfield, CT) were cultured as described by supplier. To generate Tel-Syk fusion protein, the human TEL gene (NM_001987.4) corresponding to amino acids 1–336 was cloned in-frame with the human Syk (NM_003177.5) kinase domain corresponding to amino acids 341–612 (essentially as described in [21]) and with carboxy-terminal V5/histidine tags. Tel-Syk-V5 was then subcloned into tet-operator containing pcDNA4 (Life Technologies, Grand Island, NY) for tetracycline regulation and transfection into HEK293 T-REx™ and BaF/3. LAD2 cells were obtained from the National Institutes of Health and grown as described [22]. MH7A cells were obtained from RIKEN Bioresource Center (Japan) and grown as described [23]. No clinical trials or experiments on human subjects or patients were conducted as part of this study, so ethical approval was not required.

### Antibodies and reagents

Antibodies used in this study were rabbit anti-Syk pY525/526 (Cell Signaling Technology, Beverly, MA), mouse anti-V5 and goat anti-IgM (Life Technologies, Grand Island, NY), goat anti-rabbit IR680 and goat anti-mouse IR800CW (Li-Cor, Lincoln, NE), anti-human CD3 antibody (R&D Systems, Minneapolis, MN), anti-CD28 antibody and PE labeled anti-human CD69 (BD Biosciences, San Diego, CA), AffiniPure F(ab’) fragment goat anti-human IgM and Biotin-SP conjugated goat anti-human H+L IgG (Jackson Immunoresearch, West Grove, PA), anti-IgE (Biosource, Carlsbad, CA). Growth factors used in this study were recombinant human IL-2, VEGF, TNFα, and IL-1β (R&D Systems, Minneapolis, MN), human SCF (Life Technologies, Grand Island, NY), and recombinant human GM-CSF (PeproTech, Rocky Hill, NJ). Other reagents used were RosetteSep B-cell Reagent (Stem Cell Technologies, Vancouver, BC), Ficoll-Paque Plus (Amersham GE, Piscataway, NJ), FBS Stain Buffer (BD Biosciences, San Diego, CA), and Streptavidin (VWR, Radnor, PA).

### Biochemical assays

#### Syk Homogeneous Time Resolved Fluorescence (HTRF) Assay and IC50 Determination

CC-509 was prepared at a concentration of 1.5 mM in dimethyl sulfoxide (DMSO) followed by 3-fold dilutions in Greiner 384 well polypropylene plates. A 1:4 dilution of compounds by 8μL/well transfer from the polypropylene plates to 24 μL/well Assay Buffer (50 mM 4-(2-hydroxyethyl)-1-piperazineethanesulfonic acid (HEPES) pH 7.6, 1 mM dithiothreitol (DTT), 10 mM magnesium chloride (MgCl2), 0.01% Triton X-100, 0.01% bovine serum albumin (BSA) and 0.1 mM ethylene glycol tetracetic acid (EDTA) in Costar 3710 384 well black plates was then performed. Kinase (13 μL/well of 9.62 ng/ml Syk (Carna Biosciences 08–176)) in Assay Buffer) or Assay Buffer only (background controls) were added to Costar 3710 384-well plates for the HTRF assays and compound (2 μL/well) was added from the 1:4 dilution plate. Syk start mix (87.5 μM ATP, 80 nM Substrate Peptide (American Peptide Company 332722)) was added and the mixture was incubated at room temperature for 1 hour. Syk Stop Solution (120 mM EDTA in dilution buffer) was then added to the Syk HTRF assay plate and incubated at room temperature on a shaker for 2 minutes. Syk Antibody Mix (4.86 μg/mL DyLight 647 Streptavidin (Pierce 21824); 1 μg/mL Lance Eu-Anti-Phosphotyrosine (PerkinElmer AD0069)) was added to the Syk HTRF assay plate and the mixture was further incubated at room temperature for >4h. Time resolved fluorescence was read in assay plates on a PerkinElmer EnVision. HTRF assays were performed using 320 nm excitation, 60 μs delay, 665 nm and 615 nm emission. Data was expressed by division of 665 nm by 615 nm emissions. The IC_50_ value was determined as described in the data fitting section below.

#### JAK2 HTRF assay and IC50 determination

CC-509 was prepared at a concentration of 1.5 mM in DMSO followed by 3-fold dilutions in Greiner 394 well polypropylene plates. A 1:4 dilution of compounds by 8 μL/well transfer from the polypropylene plates to 24 μL/well Assay Buffer (50 mM HEPES pH 7.6, 1 mM DTT, 10 mM MgCl2, 0.01% Triton X100, 0.01% BSA and 0.1 mM EDTA) in Costar 3573 384 well black plates was then performed. Kinase (13 μL/well of 4.6 ng/mL JAK2, Millipore 14–511) or Assay Buffer only (background controls) were added to Costar 3573 384-well black plates and compound (2 μL/well) was added from the 1:4 dilution plate. Substrate/detection mixture (10 μL/well) (30 nM DyLight 647-Streptavidin (Pierce 21824), 750 ng/mL Eu-anti-phospho-Tyrosine PerkinElmer AD0069), 37.5 μM ATP and 500 nM FLT3 (Tyr 589) Biotinylated Peptide (Cell Signaling Technology 1305)) was added and the mixture was incubated at room temperature for 1 hour. 10 μL/well of stop mixture (60 mM EDTA/0.01% Triton X-100) was then added to the JAK2 HTRF assay plate and mixed on a shaker for 2 minutes. The mixture was further incubated at room temperature for >1h. Time resolved fluorescence was read in assay plates on a PerkinElmer EnVision. HTRF assays were performed using 320 nm excitation, 60 μs delay, 665 nm and 615 nm emission. Data was expressed by division of 665 nm by 615 nm emissions. The IC_50_ value (the concentration required to inhibit enzyme activity by 50%) was determined as described in the data fitting section below.

#### Ret A HTRF assay and IC50 determination

CC-509 was prepared at a concentration of 1.5 mM in DMSO followed by 3-fold dilutions in Greiner 394 well polypropylene plates. A 1:4 dilution of compounds by 8 μL/well transfer from the polypropylene plates to 24 μL/well Assay Buffer (50 mM HEPES pH 7.6, 1 mM DTT, 10 mM MgCl2, 0.01% Triton X-100, 0.01% BSA and 0.1 mM EDTA) in Costar 3710 384 well black plates was then performed. Kinases (13 μL/well of 31 ng/ml RetA (Life Technologies, Grand Island, NY PV3819) in Assay Buffer) or Assay Buffer only (background controls) were added to Costar 3710 384-well black plates for the HTRF assays and compound (2 μL/ well) was added from the 1:4 dilution plate. RetA Substrate/Detection mix (20 μM ATP, 500 nM Gastin Precursor (Tyr 87) Biotinylated Peptide (Cell Signaling Technology 1310), 1.65 μg/ml DyLight 649 Streptavidin (Pierce 21845), and 750 ng/ml Lance Eu-Anti-Phosphotyrosine (PerkinElmer AD0069)) was added and the mixture was incubated at room temperature for 1 hour. The assay was stopped with 10 μL/well of stop solution (60 mM EDTA/0.01% Triton X-100) and then incubated at room temperature on a shaker for 2 minutes. The mixture was further incubated at room temperature for >4h. Time resolved fluorescence was read in assay plates on a PerkinElmer EnVision. HTRF assays were performed using 320 nm excitation, 60 μs delay, 665 nm and 615 nm emission. Data was expressed by division of 665 nm by 615 nm emissions. The IC50 value was determined as described in the data fitting section below.

#### KDR HTRF assay and IC50 determination

CC-509 was prepared at a concentration of 1.5 mM in DMSO followed by 3-fold dilutions in Greiner 394 well polypropylene plates. A 1:4 dilution of compounds by 8 μL/well transfer from the polypropylene plates to 24 μL/well Assay Buffer (50 mM HEPES pH 7.6, 1 mM DTT, 10 mM MgCl2, 0.01% Triton X-100, 0.01% BSA and 0.1 mM EDTA) in Costar 3573 384 well black plates was then performed. Kinase (13 μL/well of 7.7 ng/ml KDR (Life Technologies, Grand Island, NY PV3660) in Assay Buffer) or Assay Buffer only (background controls) were added to Costar 3573 384-well black plates and compound (2 μL/ well) was added from the 1:4 dilution plate. Substrate/detection mixture (10 μL/ well) (30 nM DyLight 647-Streptavidin (Pierce 21824), 750 ng/mL Eu-anti-phospho-Tyrosine (PerkinElmer AD0069), 500 μM ATP, and 500 nM Tyrosine Kinase Biotinylated Peptide Substrate 2 (Pierce 29914)) was added and the mixture was incubated at room temperature for 1 hour. 10 μL/well of stop solution (60 mM EDTA/0.01% Triton X-100) was then added to the KDR HTRF assay plate and mixed on a shaker for 2 minutes. The mixture was further incubated at room temperature for >1h. Time resolved fluorescence was read in assay plates on a PerkinElmer EnVision. HTRF assays were performed using 320 nm excitation, 60 μs delay, 665 nm and 615 nm emission. Data was expressed by division of 665 nm by 615 nm emissions. The IC50 value was determined as described in the data fitting section below.

#### Aurora A HTRF and IC50 determination

CC-509 was prepared at a concentration of 1.5 mM in dimethyl sulfoxide (DMSO) followed by 3-fold dilutions in Greiner 384 well polypropylene plates. A 1:4 dilution of compounds by 8 μL/well transfer from the polypropylene plates to 24 μL/well Assay Buffer (50 mM HEPES pH 7.6, 1 mM DTT, 10 mM MgCl2, 0.01% Triton X-100, 0.01% BSA and 0.1 mM EDTA in Costar 3710 384 well black plates was then performed. Kinases (13 μL/well of 9.62 ng/ml Aurora A (Millipore 14–511) in Assay Buffer) or Assay Buffer only (background controls) were added to Costar 3572 384-well white plates and compound (2 μL/well) was added from the 1:4 dilution plate. 10 μL/well of Aurora A Substrate/ATP mix in Assay Buffer (1.25 μM FAMPKAtide (Molecular Devices R7250 / R7255) and 12.5 μM ATP) was added and the mixture was incubated at room temperature for 1 hour. Then 60 μL/well of Binding Solution (16% 5X (80% of 1X) IMAP Progressive Binding Buffer A, 4% 5X (20% of 1X) IMAP Progressive Binding Buffer B, 1:800 Progressive Binding Reagent, and 1:200 TR-FRET Tb Donor) was added to the Aurora A IMAP TR-FRET assay and incubated overnight at room temperature. The assay plates were on the PerkinElmer EnVision. The IC50 value was determined as described in the data fitting section below.

#### Syk protein production, crystallography, and structure determination

The coding sequence for the SYK Kinase domain (residues 343–635) with a C-term 6X His tag was cloned into the pDEST8 vector using Gateway® cloning (Life Technologies, Grand Island, NY). Bacmids were generated using DH10Bac (Life Technologies, Grand Island, NY), and protein was expressed by BV transfection of Sf9 cells. SYK was purified by Ni-affinity and size exclusion chromatography. Sf9 cell pellet was resuspended in 50 mM Tris-Cl, pH 8.0, 500 mM NaCl, 10% Glycerol, 0.01% Brij-35, 1.4 mM β-mercaptoethanol, 40 mM Imidazole, EDTA-free protease inhibitor cocktail (San Diego Bioscience), 2 mM MgCl2, DNase I, and lysed by stirring at 4°C for 30min. Lysate was centrifuged at 40, 000g for 1hr and applied to a His HiTrap column. Protein was eluted with 50 mM Tris-Cl, pH 8.0, 500 mM NaCl, 10% Glycerol, 0.01% Brij-35, 1.4 mM beta-mercaptoethanol, 500 mM Imidazole with protease inhibitors. Fractions containing SYK protein were pooled and concentrated to 5-10ml using 10K MW cutoff Vivaspin concentrators, and loaded on a superdex-200 gel filtration column. Fractions containing SYK protein were pooled and concentrated to 6mg/ml in 50 mM Tris-Cl, pH 8.0, 500 mM NaCl, 10% Glycerol, 5 mM DTT. SYK was crystallized by sitting drop vapor diffusion. Briefly, SYK protein was mixed 1:1 with, and subsequently equilibrated against a reservoir solution of 100 mM MES pH 5.3, 150 mM Ammonium sulfate, and 22% PEG 3350. Ligand structures were obtained by soaking pre-grown crystals, in which compounds in DMSO were added directly to droplets containing crystals to a final concentration of 3mM for 3 hours. Crystals were cryoprotected by addition of 20% ethylene glycol and cooled under liquid nitrogen. X-ray diffraction data were collected from a single crystal under standard cryogenic conditions at the Advanced Light Source (ALS) using the ALS-5.0.2 beamline and data were integrated and scaled using HKL2000 [24]. The SYK structure was solved by molecular replacement using SYK kinase domain as a search model (PDB code 1XBA) with MOLREP. The initial molecular replacement solution was followed by iterative rounds of model building using COOT, followed by restrained refinement using REFMAC [25]. Information below provides crystallographic and refinement statistics and has been deposited in the Protein Data Bank with the accession code 4WNM. Additional technical information (numbers in parenthesis are for outer shell): Ligand: 509; Data collection site: ALS 5.0.2; Wavelength (Ǻ): 1.0; Resolution Range (Ǻ): 36.5–2.5 (2.54–2.50); Spacegroup: *P*2_1_2_1_2_1_; Cell dimensions (Ǻ): 39.87 84.82 90.62; Angles (deg): 90 90 90; No. of observations: 236298; No. of unique observations: 9994; Completeness (%):88.9 (88.6); *I / σI*: 16.01 (1.53); R_merge (%)_:0.082 (0.760); Refinement statistics: R_work_ / R_free_: 20.39 / 26.00; RMSD for bond length (Ǻ): 0.013; RMSD for bond angles (deg): 1.517.

#### Protein activity or binding panels and assays

**Fig 1:**
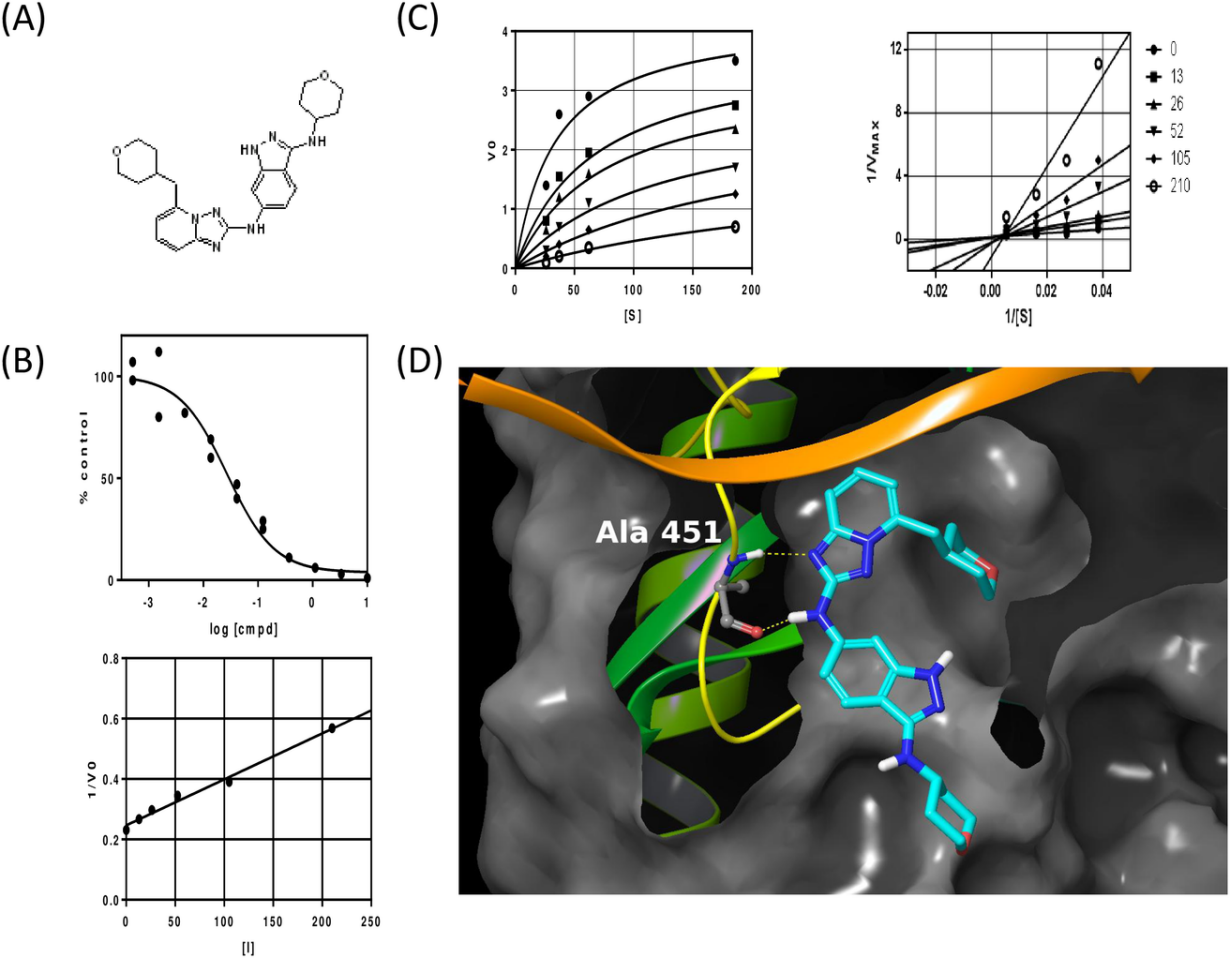
Biochemical characterization of CC-509. (A) Chemical structure of CC-509 with IUPAC name of [3-(2H-3,4,5,6-tetrahydropyran-4-ylamino)(1H-indazol-6-yl)][5-(2H-3,4,5,6-tetrahydropyran-4-ylmethyl)(2H-1,2,4-triazolo[1,5-a]pyridin-2-yl)]amine. (B) Representative IC_50_ curve (top panel) and K_i_ determination (bottom panel) for CC-509 tested against recombinant Syk enzyme. (C) Michaelis-Menten (left panel) and Lineweaver-Burk (right panel) plots for Syk enzyme tested against increasing amounts of substrate and concentrations of CC-509 (shown in nM). (D) Crystallographic diagram of Syk ATP pocket when bound with CC-509. Key residue alanine 451 and hydrogen bonds are shown.

Syk kinase mode of inhibition studies, including parameters described in Fig 1, were conducted on the LC3000 platform by Caliper Life Sciences (Hopkinton, MA) according to standard protocols developed by the supplier. For kinase activity, percent inhibition at 3 μM and IC50 determinations were measured using either the Z’-Lyte^®^ or the Adapta^®^ protocols as part of the SelectScreen^™^ profiling service (Life Technologies, Grand Island, NY). Non-kinase enzyme assays and GPCR, ion channel, and transporter binding assays were run by CEREP (France). Non-GLP manual patch clamp hERG assays were run by ChanTest (Cleveland, Ohio). Vivid^®^ Assay kits (Life Technologies) were used to measure the cytochrome P450 inhibition of the compound against CYP3A, CYP2C9, CYP2C19, CYP2D6, and CYP1A2, as described [26].

### Cellular assays

#### LAD2

Cells were cultured in StemPro-34 media (Life Technologies) with 100 ng/ml human SCF (GIBCO) and stimulated as described by supplier [22]. Briefly, LAD2 cells were gently dislodged from culture flask, added to 96-well round bottom plates, and sensitized with 0.05 μg/ml final concentration of NP-IgE (AbD Serotec, Oxford, UK) for 16 hours in a standard tissue culture incubator at 37C with 5% CO2. Cells were washed once and equilibrated in Tyrode’s Buffer (Sigma Chemicals, St. Louis, MO) for 3 hours, preincubated with CC-509 for 30 minutes, and stimulated with 0.1 μg/ml NP_16_-BSA (Biosearch Technologies, Novato, CA) for 90 minutes. Beta-hexosaminidase activity was measured in supernatants and solubilized cell pellets using p-Nitrophenyl N-acetyl-β-D-Glucosaminide substrate (Sigma Chemicals, St. Louis, MO) to generate a colorimetric signal that was measured in a SpectraMax (Molecular Devices) spectrophotometer at 405 nm. Percent release (% release) was calculated as described in Data Analysis section.

#### Basophil human whole blood assay

Assay was conducted according to the protocol for the BasoTest kit provided by supplier (Biocarta, San Diego, CA), with slight modification. Briefly, 100μl of heparinized human whole blood was added to each well of a 24-well deep plate (Microliter Analytical Supplies, Suwanee, GA) and preincubated with CC-509 for 30 minutes in a 37°C water bath. Basophils were stimulated with mite or dust extract (included in kit), or anti-IgE (diluted 1:10,000) for 20 minutes in a 37°C water bath and then on ice for 5 minutes. Cells were incubated in the FcεR receptor and CD63 antibody staining solution (included in kit) for 20 minutes on ice followed by agitation in a fixative/ red blood cell lysis buffer (included in kit) for 10 minutes at room temperature. Cells were washed twice with Basosalt solution (included in kit) and the final cell suspensions was transferred to U-bottom 96-well plates (BD Falcon, San Diego, CA) for analysis by flow cytometry on the FACSArray Bioanalyzer (BD Biosciences, San Jose, CA). Standard gating methods were used to identify basophils [27] and to measure the mean intensity of CD63 fluorescence. FCS files were exported and analyzed as described in the Data Analysis section.

#### Primary human neutrophils and eosinophils

Cells were purified from EDTA-treated human whole blood using Polymorphprep (VWR, Radnor, PA) according to the protocol provided by the supplier. Eosinophils were further purified using an Eosinophil Kit (Miltenyi, Auburn, CA) according to manufacturer’s instructions. Neutrophil and eosinophil cell pellets were incubated in phenol red-free RPMI (Life Technologies, Grand Island, NY) for 30 minutes, washed in Kreb’s Ringer buffer, and plated in 96-well white opaque plates (Perkin Elmer, Foster City, CA) for luminescence. Neutrophils were diluted with Kreb’s Ringer buffer containing luminol (200 μM final concentration, Sigma Chemicals, St. Louis, MO) and preincubated with CC-509 for 30 minutes at 37°C. Neutrophils were activated with 0.5 mg/mL final concentration of opsonized zymosan, incubated for 30 minutes at room temperature in the dark, and then read on an Envision 2104 Multilabel Reader (Perkin Elmer, Foster City, CA). Opsonized zymosan was prepared as follows: Zymosan A (Sigma Aldrich, St. Louis, MO) was boiled, washed, and resuspended to a final concentration of 20mg/ml in 150mM NaCl. An equal volume of human serum, type AB (MP Biomedicals, LLC, Santa Ana, CA) was mixed with zymosan for 30 minutes at 37°C, washed twice in 150mM NaCl, resuspended in PBS to 20mg/ml, and stored at -20°C until use. For eosinophils, cells were stimulated with IL-3 (200 pM, R&D Systems, Minneapolis, MN) for 24 hours at which point supernatants were collected and assessed for MCP-1 (Mesoscale).

#### Tel-Syk biomarker and viability assays

HEK293 T-REx™ (Life Technologies, Grand Island, NY) stably transfected with human TEL-Syk (cloned as described prior section DNA constructs) were plated on black poly-D-lysine coated 96-well plates (BD Biosciences, San Diego, CA) in culture media containing tetracycline-free fetal bovine serum (Clontech, MountainView, CA) for 16 hours in a tissue culture incubator. Tel-Syk expression was induced for 6 hours in fresh growth media containing 1 μg/ml tetracycline (Sigma Chemicals, St. Louis, MO). Plates were preincubated with CC-509 for 90 minutes, fixed immediately with 4% formaldehyde in 1X PBS for 20 min at room temperature, and washed/permeabilized 5-times with PBS containing 0.1% Triton X-100. Plates were blocked with Odyssey Blocking Buffer for 90 minutes at room temperature and incubated overnight at 4°C on a rotating platform with rabbit phospho-Syk (1:1000) and mouse V5 (1:2500) primary antibodies diluted in Odyssey Blocking Buffer. The plates were washed 5 times with 1X PBS + 0.1% Tween-20, incubated for 1 hour at room temperature with anti-rabbit IR680 (1:1000) and anti-mouse IR800CW (1:1000) secondary antibodies diluted in Odyssey Blocking Buffer, and washed 5 additional times with 1X PBS + 0.1% Tween-20 at room temperature. The plates were scanned on the Li-Cor Odyssey instrument (Li-Cor Biosciences, Lincoln, NE) in the 700 and 800 channels. Viability of BaF/3 cells expressing Tel-Syk grown in the absence of murine IL-3 was measured using Cell-Titer Glo reagent (Promega, Madison, WI), as described by supplier.

#### KDR cellular selectivity assay

HEK KDR cells were plated on 96-well flat bottom plates for 16 hours in a tissue culture incubator. Plates were preincubated with CC-509 for 60 minutes, stimulated at 37°C with 50 ng/ml final concentration of recombinant human VEGF for 5 minutes, and then processed for phospho-KDR Mesoscale assay as described by manufacturer (Mesoscale, Gaithersberg, MD). Briefly, cells on plates were lysed with Mesoscale lysis buffer (containing phosphatase/ proteinase inhibitors and 0.05% SDS), frozen at -80°C for 2 hours, and then placed on an orbital shaker at 4°C for 16 hours. Cell lysate was transferred to a blocked phopsho-KDR plate, incubated at room temperature for 2 hours, washed twice, and blotted dry. The detection antibody was added to all wells on the plate, incubated for 2 hours at room temperature on an orbital shaker, and plate was washed three times. Diluted Mesoscale detection solution was added to each well and the plates were read on the Sector Imager instrument (Mesoscale, Gaithersberg, MD).

#### JAK2 cellular selectivity assay

HEL Irf1-β-lactamase cells were harvested, plated in 384-well plates, and incubated with CC-509 at indicated concentrations for 16 hours in a tissue culture incubator. The cells were then treated with Live Blazer-FRET B/G Substrate solution (Life Technologies, Carlsbad, CA) for 6 hours at room temperature in the dark. Fluorescence readings at 460 nm and 530 nm were taken using the Flexstation II (Molecular Devices). Myeloid colony forming assays to assess Jak2 cellular activity were conducted at ReachBio (Seattle, WA).

#### Ramos NFAT β-lactamase reporter assay

Cells were assayed as described by supplier (Life Technologies, Carlsbad, CA), with slight modification. On the day prior to the assay, cells were counted, resuspended in Assay Medium at a density of 0.4 X 10^6^ cells/ml, and placed in a tissue culture incubator for 18 hours. The cells were then plated in 384-well plates and preincubated with CC-509 at indicated concentrations for 30 minutes. The cells were stimulated with 50μg/ml final concentration of anti-IgM for 5 hours in the tissue culture incubator and then treated with Live Blazer-FRET B/G Substrate solution (Life Technologies, Carlsbad, CA) for an additional 2 hours at room temperature in the dark. Fluorescence readings at 460 nm and 530 nm were taken using the Flexstation II (Molecular Devices).

#### Primary human B-cells

Buffy coat-derived purified B-cells were generated using the RosetteSep B-cell and Ficoll-Paque Plus reagents, as described in protocols provided by the suppliers. Purified B-cells were plated in 96-well U-bottom plates, preincubated with CC-509 at indicated concentrations for 30 minutes, and then stimulated with anti-IgM for 14 hours in a tissue culture incubator. Plates were washed once in FBS Stain Buffer and incubated with the anti-CD69 antibody in the dark for 30 minutes at room temperature. Plates were washed three times and analyzed by flow cytometry on the FACSArray Bioanalyzer. Standard gating methods were used to identify B-cells and to measure the mean intensity of CD69 fluorescence. FCS files were exported and analyzed as described in the Data Analysis section.

#### Primary human macrophages

Mobilized CD14-positive monocytes were plated in 96-well flat bottom plates in RPMI media supplemented with 100 ng/ml GM-CSF for 5 days. The plates were preincubated with CC-509 at indicated concentrations for 30 minutes. Biotinylated anti-IgG and streptavidin at 10 μg/ml final concentrations were thoroughly mixed, added to appropriate wells on the cell plate, and plate placed back in tissue culture incubator 8 hours. Following incubation, supernatants were collected and processed for TNFα Mesoscale single-plex cytokine assay as described by manufacturer (Mesoscale, Gaithersberg, MD). Briefly, the plates were incubated with sample or calibrator for 2 hours on an orbital shaker at room temperature. The detection antibody was added to all wells, the plate incubated for 2 hours at room temperature on an orbital shaker, and washed three times. Diluted Mesoscale detection solution was added to all wells and the plates were read on the Sector Imager instrument (Mesoscale, Gaithersberg, MD) plate reader.

#### MH7A synoviocytes

Cells were maintained in culture and assayed as described by supplier [23]. Briefly, MH7A cells were seeded to 6000 cells/ well in 96 well plates 1 day prior to stimulation. Cells were pretreated with compounds for 30 minutes, stimulated with TNFα (2 ng/ml) or IL-1β (0.5 ng/ml) and placed in tissue culture incubator for 16 hours. Supernatants were collected and assayed for cytokines using the Mesoscale Proinflammatory Panel I or Proinflammatory 4-plex according to the manufacturer’s protocol (Mesoscale, Gaithersberg, MD).

### *In vivo* studies

Animal procedures follow federal PHS policies and are approved by the Institutional Animal Care and Use Committee (IACUC) at Celgene Corporation.

#### Pharmacokinetics

Male and Female Sprague-dawley rats with jugular vein cannulation were obtained from Charles River Laboratories. All rats were maintained under standard conditions with access to food and water *ad libitum*. For intravenous (i.v.) dosing, CC-509 was dissolved in DMA/PEG400 (15/85, V/V). A dose of 2 mg/kg was administered to three male rats via jugular vein cannula with a dosing volume of 2 mL/kg followed by flushing 0.5 mL saline through the cannula. Blood were collected at 0.083, 0.25, 0.5, 1, 2, 4, 6, 8 and 24hr post dose. Plasma samples were separated by centrifugation of blood and stored at -20°C until analysis. For oral administration, four female rats (220–250g) were fasted for 12hr but allowed to access water prior to the dosing. CC-509 was formulated in 20% Captisol as a solution and was administered to the rats at 10 mg/kg by oral gavage with a dosing volume of 5mL/kg. Food was returned to the rats 2hr post dosing. Blood samples were collected at 0.5, 1, 2, 4, 6, 8 and 24hr post dose. Plasma samples were separated by centrifugation of blood and stored at -20°C until analysis. Plasma concentrations of CC-509 were quantified by a LC/MS/MS method. A total of 50 microliters (μL) of each plasma sample was extracted with three volumes of mixed solvent (methanol:acetonitrile (1:1). The extracted samples were filtered through a filtration plate (0.45μm, Varian, Inc.) and the filtrates were injected onto a LC-MS system. The LC-MS system consisted of a ThermoScientific (Waltham, MA) TSQ Quantum Ultra™ Mass Spectrometer coupled with a Thermo Accela™ ultra-high pressure liquid chromatogram (UPLC) system. Separation was achieved on a Thermo Hypersil Gold™ column (50 x 2.1 mm, 1.9 μm) by running a linear gradient of mobile phase at a flow rate of 0.5 mL/min. Mobile phase A and B consisted of 0.1% formic acid in water and 0.1% formic acid in methanol, respectively. Quantitation was performed using selected reaction monitoring (SRM) at the transition m/z 448 to m/z 364 for CC-509. PK parameters in plasma were estimated by WinNonLin software (Pharsight, Princeton, NJ) using non-compartmental analysis. For i.v. dosing, plasma clearance (CLp), volume distribution at steady-state (Vss) and mean residence time (MRT) were estimated. For oral administration, the maximum plasma concentration (Cmax), the time when the maximum plasma concentration is reached (Tmax) and area under the curve from 0 to infinity (AUC_0-inf_) were calculated.

#### Passive cutaneous anaphylaxis

On day 0, male CD-IGS rats (170 – 190g; Charles River Laboratories) were anesthetized with isofluorane, their dorsal region shaved and 50 μl Anti-DNP-IgE (Clone SPE-7, Sigma, St. Louis, MO) that had been diluted 1:1000 in saline was injected via the intradermal route at 3 individual spots along the right side of the back. Saline was injected via the intradermal route at 3 individual spots along the left side of the back as a sham control. Twenty-four hours later, 200 μl of BSA-DNP (Calbiochem, San Diego, CA), prepared (1 mg prepared in a 2% solution of Evans blue dye: Sigma Chemicals, St. Louis, MO) was injected via the IV route via the tail vein. Thirty minutes after the BSA-DNP challenge the experiment was terminated and skin samples from the middle of each injection site taken for Evans blue dye measurement at an OD measurement at 620nm on a plate reader following incubation (2h at 90°C) and homogenization in formamide (Sigma Chemicals, St. Louis, MO). Vehicle (20% Captisol^®^ (Captisol), PO), the positive control (Cromolyn: Sigma Chemicals, St. Louis, MO 100mg/kg, 2mL/kg, IV) or CC0484509 (3 and 10mg/kg, PO) were dosed 1h prior to the IV challenge with BSA-DNP.

#### Collagen-induced arthritis

Female Lewis rats (150 – 170g; Charles River Laboratories) were house standard shoebox cages, fed Harlan-Tekald diet and water ad libitum and maintained on a 12h light/dark cycle. For induction of arthritis rats were anesthetized with isofluorane and their entire dorsal area shaved. Rats were injected (intradermal) with 100 μl of equal volumes of collagen (Chrondrex, Redmond, WA) and IFA (Sigma Chemicals, St. Louis, MO) emulsion in 10 sites across the dorsal region. At disease onset 11 days following immunization animals were randomized into groups based on their ankle measurements and test article administration was initiated. Rats were dosed orally with vehicle (20% Captisol®), Indomethacin (Sigma Chemicals, St. Louis, MO, 2mg/kg) or CC-509 (15, 25mg/kg QD or 15mg/kg BID) in a dose volume of 5ml/kg. Paw swelling was assessed by caliper measurements on day 11, 12, 13, 14, 15 and 18 post-immunization and % inhibition calculated at day 18 compared to the vehicle control or area under the curve (AUC) calculated from day 11–18. On the last day of the study animals were split into 2 cohorts and bleed twice via tail bleed under isofluorane anesthesia and a third terminal bleed following CO_2_ asphyxiation to obtain a time course of plasma compound exposure at 30minutes, 1, 2, 4, 8 and 24h after the last dose of CC-509.

#### Inflammatory cytokines measured in the paw

Following the last administration of CC-509, paw samples were collected and processed for cytokine and chemokine analysis (Aushon Biosciences, Billerica, MA). Briefly paws were snap-frozen and pulverized in liquid nitrogen followed by addition of cell signal lysis buffer in the presence of protease and phosphatase inhibitors (Sigma Chemicals, St. Louis, MO). Samples were homogenized on high speed for 30 seconds, incubated on ice for 20 minutes then sonicated at high intensity for 1 minute. Samples were spun at high speed and supernatants were collected for cytokine analysis.

### Data and statistical analysis

For the TEL-Syk assay, the intensity values for the 700 and 800 channel were exported as text files in ActivityBase (IDBS, Guildford, UK) to derive the ratios that reflect kinase activity. For LAD2 experiments, enzyme activity values were exported as text files into ActivityBase to calculate “percent release” according to the formula: 100* supernatant OD405/ (supernatant OD405 + (pellet OD405*pellet dilution factor)). For the basophil and B-cells assays, flow cytometry data was exported from the FACS Array and processed using FloJo software package (TreeStar, Inc., Ashland, Oregon). For the JAK2 HEL and Ramos NFAT β-lactamase assays, the fluorescence values obtained at 460 nm and 530 nm were exported into ActivityBase to calculate response ratios. For the neutrophil, macrophage, KDR, and JAK1/3 assays, raw values were exported into ActivityBase to calculate percent inhibition. All IC50 calculations were generated using XLfit (IDBS, Guildford, UK) algorithm 205 within Excel. For PCA, the OD from the saline injected site was subtracted from the OD from the anti-DNP IgE injected site. Evans blue dye extravasation was expressed as mean ± SEM and % inhibition for the test article groups was normalized to the cromolyn positive control. For the CIA model paw measurements (left and right ankle diameter) were taken for each individual animal and averaged. The mean data from each group was plotted and either the AUC_(day 11–18)_ or % inhibition on day 18 was calculated. Data is expressed as mean ± SEM of each group with n = 6–12. Statistical analysis was performed with Prism Graphpad using one-way ANOVA followed by Dunnett’s comparison using the vehicle group as the control or for measurements over time a 2 way ANOVA followed by a Dunnett’s multiple comparison test. Statistical significance was deemed as * = p<0.05, **p<0.01, ***p<0.001 and p<0.0001. For biochemical assays, the nonlinear, least squares fitting program Xlfit4 Excel Add-in was used to fit the CC-509 dose response curves to a 4-parameter logistic model with the zero percent and 100 percent enzyme activity fixed and locked (effectively reducing to a 2-parameter fit):

$$\%Act.=\frac{100\%}{1+{\left(\frac{I}{IC_{50}}\right)}^{n}}$$

where % Act. is percent enzyme activity remaining, [I] is CC-509 concentration, IC_50_ is the concentration of CC-509 calculated to give 50% remaining enzyme activity, and *n* is the Hill slope.

## Results

### Identification and biochemical characterization of CC-509

Structure-based guided optimization of a triazolopyridine screening hit led to the identification of CC-509 (Fig 1A), an active and reversible inhibitor of Syk enzyme with an IC_50_ value = 0.026 +/- 0.006 μM (n = 8) and a Ki of 18 nM (Fig 1B). Based on enzyme kinetics, CC-509 is a mixed competitive inhibitor with respect to the Syk ATP binding site (Fig 1C). Consistent with this mode of inhibition, the co-crystal structure of CC-509 in Syk shows the inhibitor bound primarily in the ATP binding pocket via two hydrogen bonds with hinge residue Alanine 451 (Fig 1D).

To assess its overall kinome selectivity, CC-509 was initially profiled at 3 μM against a commercial panel of 256 kinases. In this format, CC-509 inhibited the activity of 27 of 256 kinases by >80% (S1 Table). Based on its activity in this panel of kinases, the calculated Gini score (a metric for kinase selectivity [28]) of 0.53 for CC-509 indicates the compound is considered moderately selective. IC_50_ values were determined for a selected subset of the kinases that were active in the initial profile (Table 1). The kinases within 3-fold potency to Syk were Flt4, Fms, and Jak family members Jak1, Jak2, Jak3. For comparison, R406 was tested under the same conditions and was shown to inhibit 75 of 256 kinases (S2 Table). CC-509 was also profiled against other potential targets to understand its broader selectivity. In a non-kinase 42 enzyme panel, CC-509 at 10 μM displayed >70% inhibition of enzyme activity against the three targets PDE2A (89%), PDE5 (90%), and 5-lipoxygenase (73%). In a panel of 80 receptors, ion channels, and transporters, CC-509 at 10 μM inhibited activity >50% in binding assays for Adenosine A1 (89%), Adenosine A2a (96%), and Adenosine A3 (84%). CC-509 also did not inhibit human ether-à-go-go-related Gene (hERG) channel activity nor the activity of any of the major Cyp450s, including Cyp1A2, Cyp2C9, Cyp2C19, Cyp2D6, Cyp3A4 (data not shown). Overall, this data suggests CC-509 is a relatively selective inhibitor across the kinome and interacts with a restricted number of other potential cellular targets.

**Table 1:** Biochemical IC50 values for CC-509 against selected human kinases.

Kinase	IC50 (μM)
*Internal data*^*a*^	
Syk	0.026 ± 0.006
KDR	0.15 ± 0.03
Jak2	0.019 ± 0.002
Ret	0.75 ± 0.19
Aurora A	0.22 ± 0.05
Fms	0.39 ± 0.31
*External data*^*b*^	
Aurora B	0.41
Flt3	0.14
Flt4	0.05
Jak1	0.04
Jak3	0.02
PDGFR-alpha	0.23
Tyk2	0.11

^a^ Data generated in-house, Mean ± SD

^b^ Data generated by commercial vendor

### CC-509 inhibits Syk in engineered cells systems

**Fig 2:**
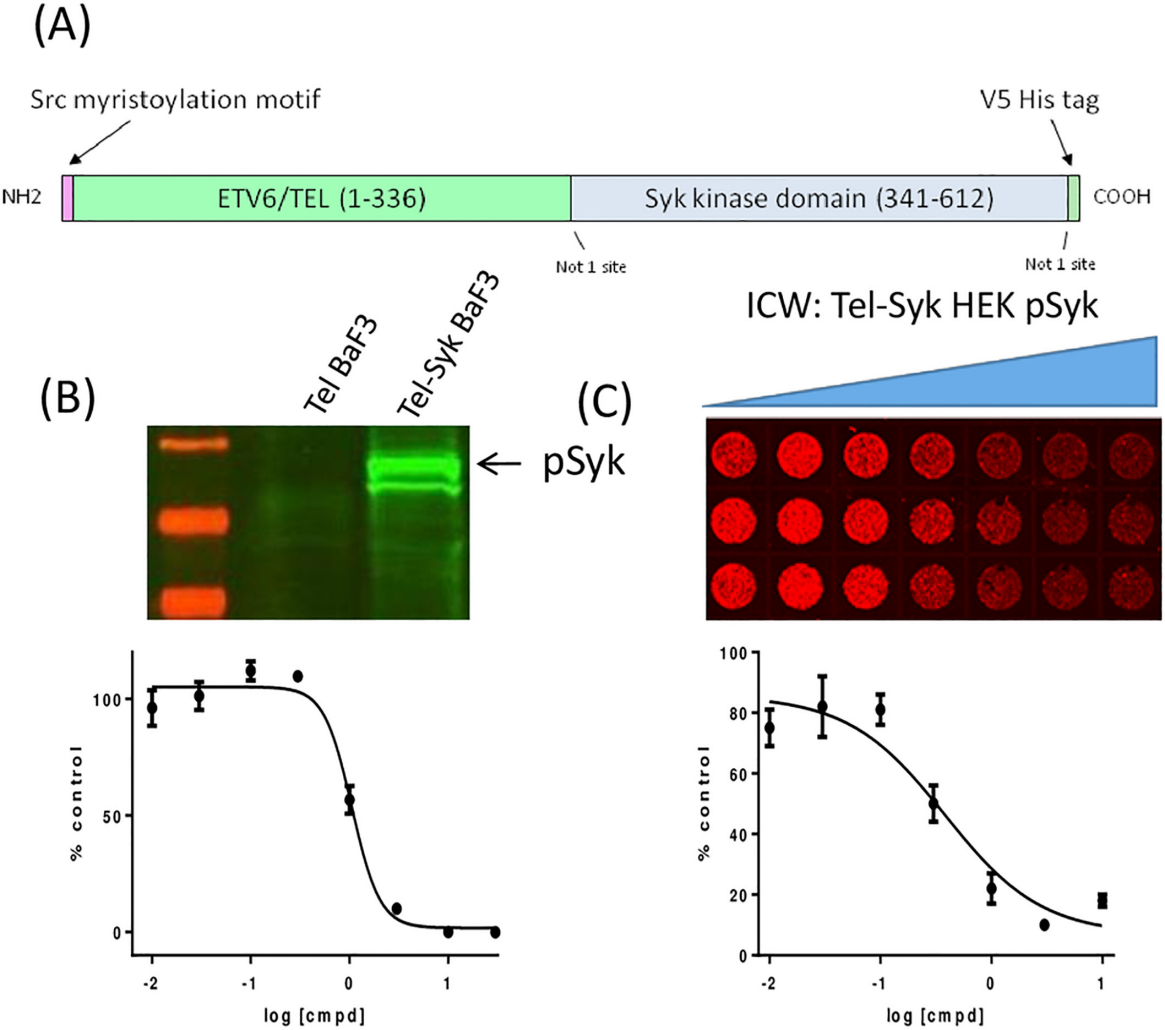
CC-509 blocks Tel-Syk cellular activity. (A) Design of human Tel-Syk fusion construct. Indicated are amino-terminal myristoylation motif, major Tel and Syk domains (amino acid numbers of each are shown), Not I cloning sites, and carboxy-terminal V5/ His tag. (B) *Top panel*: Western blot displaying Syk phosphorylation (Y525/Y526) in Tel-Syk or Tel- expressing BaF3 cells. *Bottom panel*: CC-509 inhibits growth factor-independent growth in Tel-Syk expressing BaF3 cells. Data expressed as percent DMSO-treated control, mean ± SD. (C) *Top panel*: In-cell western (ICW) image showing CC-509 inhibition of Tel-Syk dependent Syk phosphorylation (Y525/Y526) in Hek cells. Concentration of CC-509 increasing from left to right. *Bottom panel*: Quantation of top panel. Data expressed as percent DMSO-treated control, mean ± SD.

Having demonstrated that CC-509 is a potent enzymatic inhibitor of Syk, its cellular activity was first tested in engineered cell models. BaF3 cells are a murine leukemic cell line normally dependent on interleukin-3 (IL-3), but will grow independently of IL-3 when engineered to overexpress a transforming oncogene [21]. Indeed, the overexpression of a Tel-Syk fusion oncogene (Fig 2A) is sufficient to promote growth factor-independent growth in BaF3 (data not shown). Tel-Syk is a naturally occurring fusion protein found in myelodysplastic syndrome t(9;12)(q22;p12) patients in which the oligomerization motif from the Tel gene is fused in-frame with the kinase domain of Syk [29]. The close apposition of the Syk kinase domains afforded by Tel oligomerization motifs promotes the phosphorylation of Syk at tyrosine 525 and 526 (Y525/526) within the active site and drives its oncogenic activity (Fig 2B). Tel-Syk rendered catalytically inactive through active site lysine-to-methionine (K395M) mutation does not promote growth factor-independence nor Y525/526 phosphorylation (data not shown). CC-509 inhibits (IC_50_ = 1.61 ± 0.41 μM) growth factor-independent growth in Tel-Syk BaF3 (Fig 2B). The commercially available Syk II inhibitor [30] similarly inhibited the growth of Tel-Syk BaF3 (IC_50_ = 2.24 ± 0.29 μM). When the Tel-Syk fusion protein was expressed in HEK293 cells, high levels Y525/526 phosphorylation were also observed (Fig 2C) and could be blocked with CC-509 (IC_50_ value = 0.61 ± 0.19 μM). For comparison, R406 also blocked Y525/526 phosphorylation at comparable potency (IC_50_ value = 0.72 ± 0.66 μM). This data indicate CC-509 is a cell-permeable and potent inhibitor of cellular Syk kinase activity.

### CC-509 inhibits FcR-dependent cellular signaling

**Fig 3:**
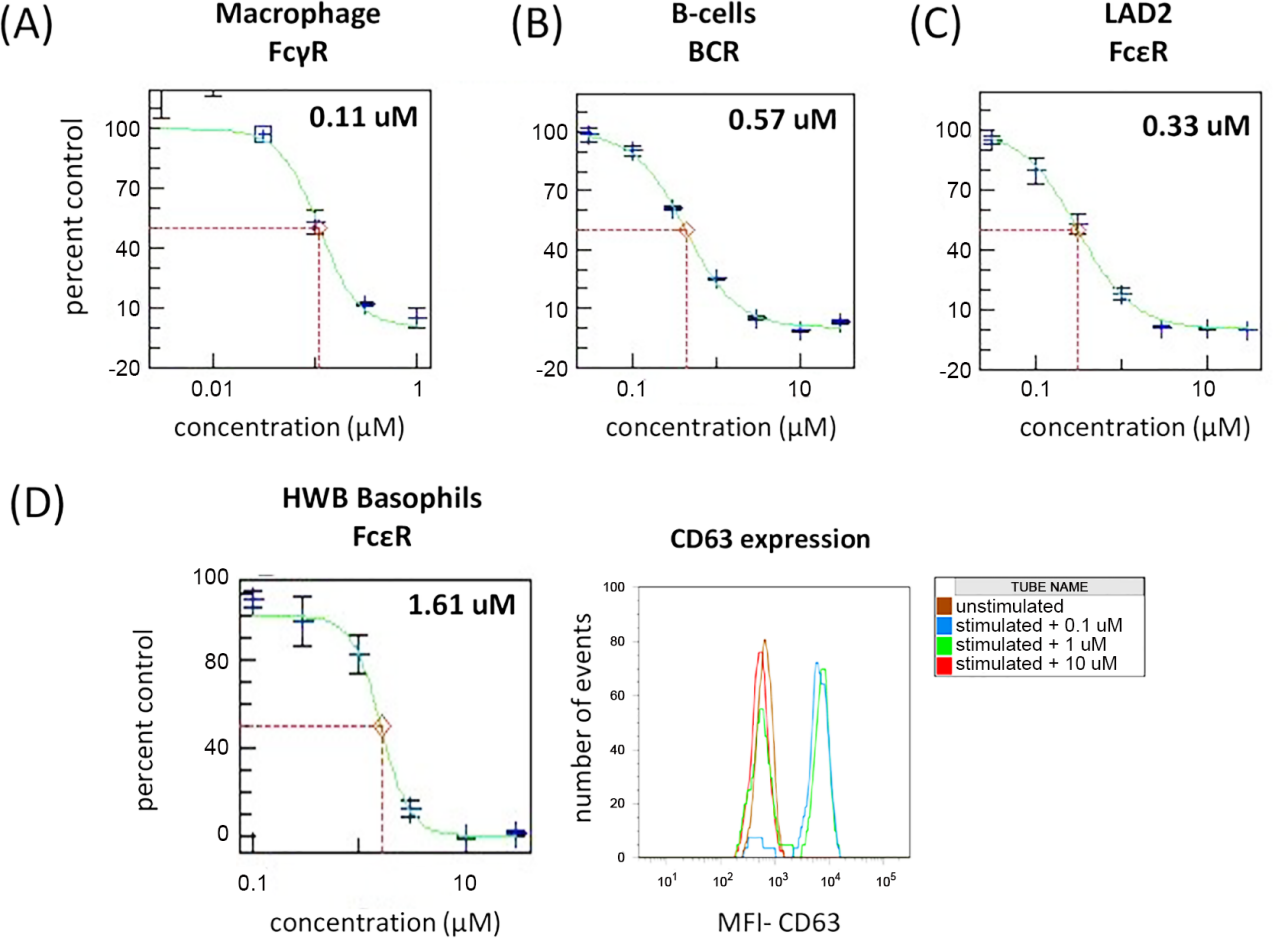
FcR-dependent functional activity in primary human immune cells is inhibited by CC-509. When stimulated through the indicated FcR, CC-509 inhibits functional activity in (A) primary human macrophage, (B) primary human B-cells and (C) the LAD2 human mast cell line. (D) *Left panel*: CC-509 inhibits CD63 upregulation on basophils stimulated through indicated FcR in human whole blood. *Right panel*: Representative flow-cytometry plot showing the number of events and CD63 mean fluorescence intensity (MFI) on unstimulated (brown trace) or stimulated basophils in the presence of 0.1 μM (blue trace), 1.0 μM (green trace), or 10.0 μM (red trace) CC-509. IC_50_ values (μM) are indicated in upper-right portion of each panel. All data expressed as percent of DMSO-treated control, mean ± SD.

The activity of CC-509 was next examined in disease-relevant cell systems in which Fc-receptors and Syk are endogenously expressed, focusing initially on primary cells from the innate and adaptive immune systems. Studies in macrophage from syk (-/-) mice or following treatment with Syk kinase inhibitors indicate Syk is coupled to and required for activation through FcγR [8,15]. Consistent with these observations, CC-509 potently inhibited TNFα production induced by FcγR cross-linking in primary human macrophage (IC_50_ = 0.17 ± 0.12 μM; Table 2, Fig 3A). R406 was also tested in this assay and exhibited roughly equivalent potency (IC_50_ = 0.29 ± 0.16 μM). BCR-dependent B-cell activation is reduced in syk (-/-) mice and can be blocked with Syk kinase inhibitors [11,15]. CC-509 potently blocked BCR-dependent upregulation of cell surface CD69, an early marker of lymphocyte activation [31], on purified primary B-cells (IC_50_ = 0.51 ± 0.09 μM; Table 2, Fig 3B). BCR-mediated cellular activity was also tested in a Ramos lymphoma cell line engineered to carry an NFAT-dependent reporter gene. BCR-mediated reporter activation in the Ramos NFAT cell line is also potently inhibited by CC-509 (IC_50_ = 0.19 ± 0.04 μM; Table 2). R406 was also active in these BCR-dependent assay systems, showing similar potency to CC-509 in both the primary B-cell assay (IC_50_ = 0.40 ± 0.22 μM) and Ramos reporter cell line (IC_50_ = 0.12 ± 0.08 μM).

**Table 2:** CC-509 inhibitory activity in immunoreceptor-dependent cellular assays.

Cellular assay	Receptor	IC50^a^
primary human B-cells^b^	BCR	0.51 ± 0.09
Ramos NFAT-*bla*^c^	BCR	0.19 ± 0.04
primary human macrophage^d^	FcγR	0.17 ± 0.12
LAD2 cells^e^	FcεR	0.22 ± 0.13
HWB Basophils ^f^	FcεR	1.52 ± 1.05

^a^Mean ± SD, μM, minimum n = 3

^b^anti-IgM stimulated CD69 upregulation

^c^anti-IgM stimulated NFAT-dependent reporter gene activity

^d^anti-IgG stimulated TNF secretion

^e^IgE-dependent beta-hexosaminidase release

^f^IgE-dependent CD63 upregulation on human whole blood basophils

The activity of CC-509 was also assessed in FcεR-dependent cell systems. The human LAD2 mast cell line expresses FcεR and can be stimulated through this receptor to release β-hexosaminidase from secretory granules [22]. CC-509 potently inhibited IgE-mediated β-hexosaminidase from LAD2 cells (IC_50_ = 0.22 ± 0.13 μM; Table 2, Fig 3C). In contrast and consistent with the position of Syk upstream from intracellular calcium stores, Syk inhibition did not block ionomycin triggered β-hexosaminidase release from LAD2 cells (data not shown). R406 was consistently more potent (IC_50_ = 0.11 ± 0.07 μM) in the LAD2 assay than CC-509, perhaps reflecting its original optimization as an inhibitor of mast cell activity [15]. CC-509 was also tested against basophils in human whole blood that were selectively stimulated through FcεR. In this assay, the degree of basophil activation is indicated by cell surface CD63 expression and quantitated by flow-cytometry [32]. CC-509 inhibited FcεR-dependent basophil activation in human whole blood (IC_50_ = 1.52 ± 1.05 μM; Table 2, Fig 3D), albeit at higher concentrations than in the LAD2 assay. Interestingly, after multiple attempts R406 was not active in the human whole blood basophil assay. FcεR-independent basophil activation using *N*-formyl-L-methionyl-L-leucyl-L-phenylalanine was not affected by CC-509 (data not shown). CC-509 inhibits FcεR-, FcγR-, and BCR-mediated signaling in disease-relevant and primary cell types.

### CC-509 activity in cellular systems not dependent on FcRs

To better understand the breadth of its cellular activity, CC-509 was also tested through modes of stimulation that did not involve FcRs but may still be dependent on Syk and again contrasted its activity to R406. In primary human neutrophils, CC-509 inhibited the release of superoxide anions following stimulation with opsonized zymosan (IC_50_ = 3.27 ± 1.27 μM), and did so less potently than R406 (IC_50_ = 1.04 ± 0.45 μM). Zymosan can promote the release of superoxide anions from neutrophils or macrophage primarily through the complement or dectin-1 receptors in a Syk-dependent manner and can induce inflammatory arthritis in rodents [33–35]. When tested in primary human eosinophils, however, CC-509 blocked the secretion of MCP-1 from IL-3 stimulated primary human eosinophils (IC_50_ = 0.11 ± 0.05 μM) much more potently that did R406 (IC_50_ = 1.41 ± 1.73 μM). Syk has also been linked to IL-3 and IL-3 receptor signaling [36]. Since synovial fibroblasts are key effector cells in RA and Syk has been implicated in their activation [37,38], we also examined the effect of CC-509 in the RA patient-derived synovial fibroblast cell line, MH7A. R406 inhibited TNFα-induced IL-6 production from MH7A (IC_50_ = 1.04 ± 0.13 μM), consistent with results in primary patient-derived synovial fibroblasts [37]. In contrast, CC-509 did not inhibit the production of IL-6 following TNF stimulation in MH7A (data not shown). This data indicate that CC-509 has cell specific effects following various modes of stimulation and provides further evidence for a differentiated profile when compared to R406.

### CC-509 activity in off-target cellular systems

Hypertension and neutropenia, two of the major side-effects observed in fostamatinib (R406) RA clinical trials, have been linked to inhibition of KDR and Jak, respectively [39,40]. R406 has comparable binding affinities to Jak2, Syk, and KDR enzymes (Jak2>Syk>KDR, each within 5-fold of Syk) [41]. Since CC-509 also displayed activity against these enzymes (see Table 1), it was run in KDR- and Jak2-based cellular assays to assess its off-target cellular activity. Consistent with its activity against the KDR enzyme, CC-509 displayed modest inhibition of VEGF-induced human KDR autophosphorylation in Hek cells (IC_50_ = 7.43 ± 3.36 μM). R406 was 10-fold more potent in the KDR cellular assay (IC_50_ = 0.72 ± 0.46 μM), indicating it is more active than CC-509 against the KDR target. In a Jak2-dependent cellular system, CC-509 was active (IC_50_ = 5.45 ± 0.59 μM) while R406 was slightly more potent (IC_50_ = 3.06 ± 1.42 μM). To further assess its activity against Jak and its potential for myeloid depletion as has been done with other Jak inhibitors [42,43], CC-509 was tested in myeloid *in vitro* colony forming assays. CC-509 partially inhibited the formation of myeloid colonies at moderate concentrations (IC_50_ = 2.8 μM) and completely inhibited colonies at a high concentration (50 μM). R406 partially inhibited myeloid colonies more potently (IC_50_ = 0.88 μM) and completely inhibited colonies at 5-fold lower concentration (10 μM) than CC-509. Taken together, these studies indicate CC-509 has moderate activity in the KDR- and Jak2-dependent cellular assays, and in all cases less activity than R406.

### CC-509 displays favorable pharmacokinetic properties in rat

The pharmacokinetic properties of CC-509 were assessed in rats to support dose selection in efficacy models. Following intravenous administration of 2 mg/kg CC-509 in male rats, the calculated PK parameters included clearance (CL = 18.9 ± 3.5 mL/min/kg), volume of distribution (V_ss_ = 2.80 ± 2.36 L/kg), area-under-curve (AUC_0-inf_ = 4.02 ± 0.70 μM·hr), and mean residence time (MRT = 2.3 ± 1.5 hr). When dosed orally at 10 mg/kg in female rats, CC-509 exhibited maximal plasma concentration (C_max_ = 4.4 ± 0.2 μM) at 30 minutes and moderate area-under-curve (AUC_0-inf_ = 12.0 ± 1.0 μM·hr). CC-509 was therefore deemed to have acceptable PK properties and was progressed into *in vivo* efficacy studies.

### CC-509 is efficacious in antibody-dependent models of inflammation and arthritis

**Fig 4:**
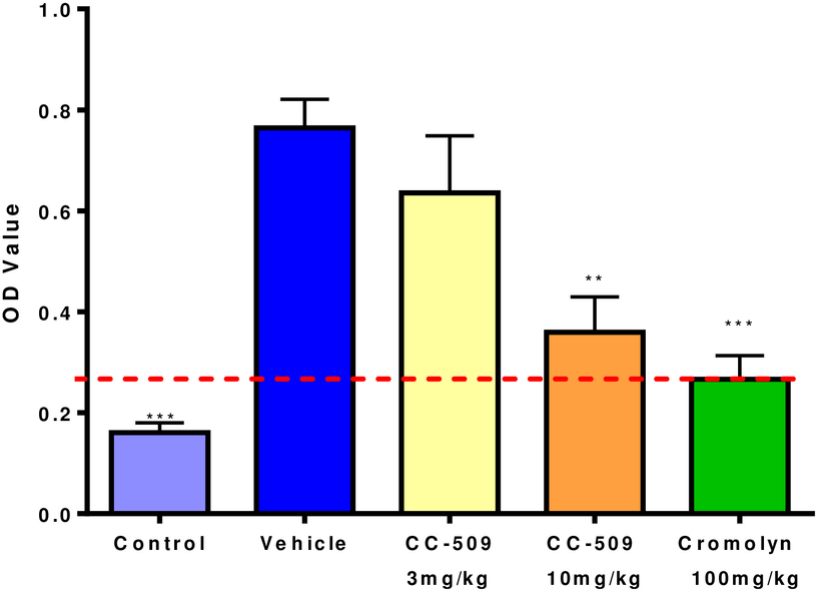
CC-509 inhibits IgE-mediated skin edema in the rat passive cutaneous anaphylaxis model. Optical density (OD) value of Evans blue dye extravasation in skin measured after immune complex challenge in untreated rats or those challenged and dosed with vehicle, 3 or 10 mg/kg CC-509, or cromolyn. Data expressed as mean ± SEM. Dashed red line, based on cromolyn, is considered 100% inhibition and was used to normalize the CC-509 data. Statistical significance at **p<0.01, ***p<0.001.

Having demonstrated it is a potent inhibitor of Syk with acceptable pharmacokinetic properties, CC-509 was tested in rodent models of inflammation and autoimmunity. Passive cutaneous anaphylaxis (PCA) is a model of inflammation in which immune complexes mediate localized vascular leakage in skin [44]. Rats were dosed orally with compound 1 hour prior and euthanized 30 minutes after immune complex challenge. The mast cell stabilizer cromolyn significantly inhibited skin edema (79%, p<0.001) compared to vehicle control group (Fig 4). The values for cromolyn were considered 100% inhibition and used to normalize the Syk inhibitor groups. CC-509 inhibited skin edema in a dose-dependent fashion when administered at 3 and 10 mg/kg, yielding 26% and 81% inhibition, respectively, and of which only the 10 mg/kg dose group was statistically significant (p<0.01) (Fig 4). In previously published studies, R406 exhibited a statistically significant effect at a 10 mg/kg dose when run in the PCA model [16]. These results indicate that CC-509 can reduce localized inflammation caused by immune complexes.

**Fig 5:**
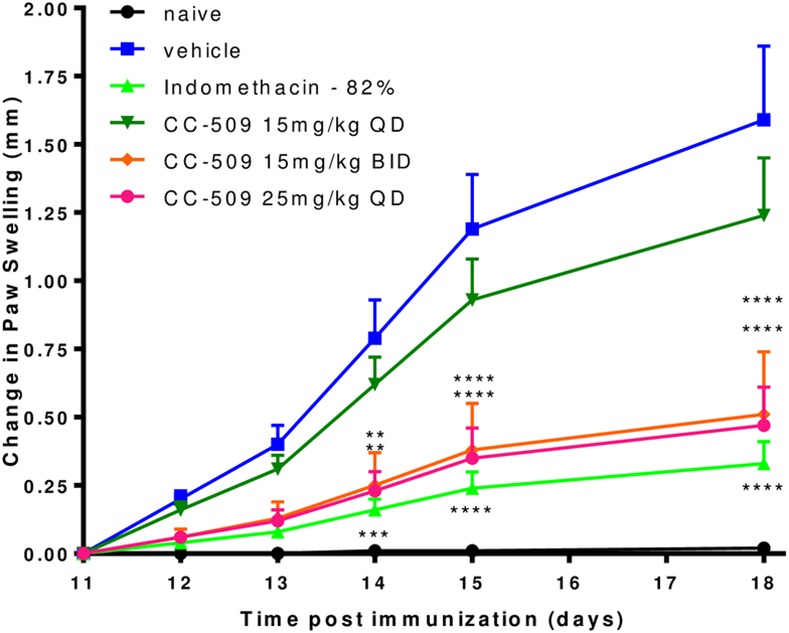
CC-509 inhibits paw swelling in rat collagen-induced arthritis model. Change in paw swelling from study day 11 to day 18 in non-immunized rats (black line) or collagen-immunized rats dosed with vehicle (blue line), indomethacin (light green line), or CC-509 at 15 mg/kg q.d. (dark green line), 15 mg/kg b.i.d. (orange line), or 25 mg/kg q.d. (red line). Data points are mean ± SEM. Statistical significance at **p<0.01, ***p<0.001, and ****p<0.0001.

Since Syk inhibitors have previously been shown to be protective in models of arthritis [16,17,45], CC-509 was tested in the collagen-induced arthritis (CIA) model. Immune complexes are an important driver of disease pathology in the CIA model [46]. In this model, rats were immunized on day 1, compound dosing and paw measurements were initiated on day 11, and the study completed on day 18. The paw measurements at day 11 ranged from diameters of 6.49 to 6.85 mm and reached an average of 8.2 ± 0.3 mm in the vehicle-treated group at the end of the study. CC-509 was administered orally for the 8 day dosing period at 15 mg/kg q.d., 15 mg/kg b.i.d., and 25 mg/kg q.d.. The 15 mg/kg b.i.d. and 25 mg/kg q.d. groups inhibited paw swelling at 72% and 74%, respectively, while 15 mg/kg q.d. inhibited at 32% (Fig 5). The effect on paw swelling was statistically significant only in the 25 mg/kg q.d. (p<0.0001) and 15 mg/kg b.i.d. (p<0.001) groups, but not in the 15 mg/kg q.d. group (Fig 5). In prior published work, R406 significantly reduced the clinical paw score in rat CIA when dosed at 10 or 30 mg/kg b.i.d., leading to exposures of 15 μM·hr and 38 μM·hr, respectively, following a single dose [16]. The non-steroidal anti-inflammatory drug indomethacin also significantly inhibited paw swelling (82%, p<0.01) (Fig 5). CC-509 exposure levels in plasma after the last day of dosing in the 15 mg/kg q.d., 15 mg/kg b.i.d., and 25 mg/kg q.d. groups were AUC_0-24h_ of 22, 60, 45 μM·hr, respectively. Within this experiment, 45 μM·hr represents the minimal efficacious exposure and, based on its activity in cellular assays, we estimate this level of CC-509 will at least partially inhibit Syk for approximately 12 hours.

**Fig 6:**
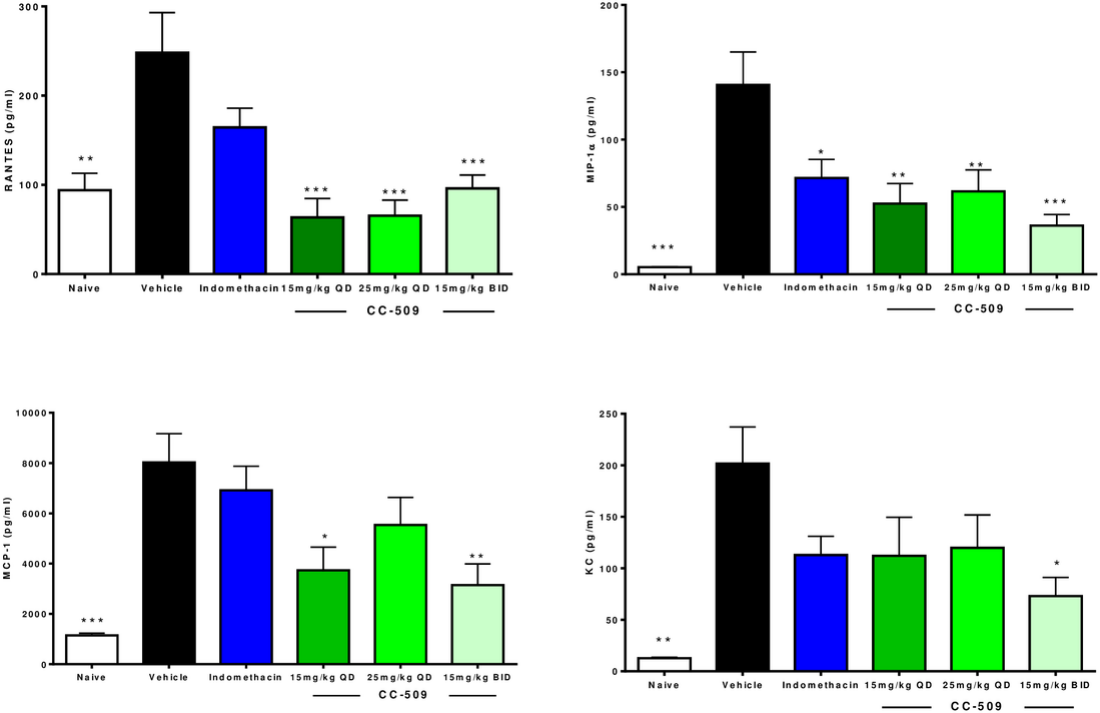
CC-509 inhibits proinflammatory chemokine production in rat collagen-induced arthritis model. Proinflammatory chemokine (clockwise from upper right: MIP-1α, KC, MCP-1, RANTES) levels on day 18 in paw tissue from non-immunized rats (white bars) or collagen-immunized rats dosed with vehicle (black bars), indomethacin (Indo, blue bars), or CC-509 at 15 mg/kg q.d. (dark green bars), 15 mg/kg b.i.d. (light blue bars), or 25 mg/kg q.d. (light green bars). Data points are mean ± SEM. Statistical significance at * = p<0.05, **p<0.01, ***p<0.001.

Following the last administration of CC-509 on day 18, paw samples were collected to measure proinflammatory analytes including MIP-1α, RANTES, IL-1β, IL-6, CRP, KC, MCP-1. All of these analytes were significantly elevated (p<0.05) in the vehicle control group paws relative to the naïve group (data not shown, Fig 6). RANTES (p<0.001) and MIP-1α (p<0.01) were significantly reduced in the paw by CC-509 at all dose levels (Fig 6). MCP-1 was significantly reduced in the 15 mg/kg q.d. and b.i.d. groups (p<0.01), while KC was reduced only in the 15 mg/kg b.i.d. group (p<0.05) (Fig 6). No other significant effects of CC-509 were observed. R406 similarly inhibited the expression of MCP-1 and KC in the CIA model, but also uniquely inhibited IL-6 and IL-1β [16]. Indomethacin significantly inhibited only MIP-1α (p<0.05). Taken together, these *in vivo* findings indicate that CC-509 can selectively inhibit inflammation and inflammatory mediators in models of autoimmune disease.

## Discussion

Syk is an established drug target due to its extensive target validation, chemical tractability, clinical data, and low potential for major on-target safety issues. In this study, we describe the identification and characterization of a novel triazolopyridine-based Syk kinase inhibitor, CC-509. The compound is a reversible, mixed ATP competitive and potent inhibitor of Syk enzymatic activity. CC-509 is also active in cellular assays, including two engineered systems in which the Syk fusion oncogene Tel-Syk is overexpressed and promotes high levels of Syk phosphorylation. Moreover, CC-509 potently inhibits FcεR-, FcγR-, or BCR-mediated signaling in disease-relevant human primary cells and in related cell lines. CC-509 exhibited potency in both neutrophil and eosinophil cellular assays, but did not have activity in the human synovial fibroblastic cell line MH7A. The robust activity of CC-509 in eosinophils is unexpected and should be followed-up in models of asthma or eosinophilia. CC-509 has a clearly diferentiated cellular profile when compared directly to R406 in these same assays. In addition, CC-509 displays favorable PK properties in rodents, including oral bioavailability and evidence for acceptable target coverage. In the immune complex-mediated PCA model of inflammation, CC-509 inhibits tissue edema measured through dye extravasation in a dose-dependent fashion, with an ED50 of approximately 5 mg/kg. Similarly, CC-509 reduced paw swelling in a dose-dependent manner and pro-inflammatory cytokine production in the rat CIA model with an ED50 of approximately 10 mg/kg. When comparing *in vivo* efficacy of CC-509 to R406, the exposure (when accounting for R406 b.i.d. dosing) and size of effect at minimal efficacious dose are roughly similar, although differences in cytokine inhibition are observed. Our studies provide additional support for the role of Syk in immune cell modulation and the use of Syk kinase inhibitors as disease modifying drugs, and indicate CC-509 is differentiated from R406.

While Syk is clearly a mediator of immune cell signaling, it may also be a driver of disease in a number of inflammatory and autoimmune conditions such as RA or Lupus. For example, in RA, fibroblast-like synoviocytes exhibit significantly higher levels of activated Syk than comparable cells from osteoarthritis patients or normal individuals and display reduced TNFα-dependent signaling following treatment with R406 [37]. We observed similar results with R406 in the human RA-derived synoviocyte cell line MH7A (see text). Moreover, Syk pathway genes are upregulated in synovial tissues from CIA-susceptible rodents when compared to CIA-resistant congenic controls [47]. In the context of lupus, T-cells from a subset of patients display aberrant signaling due to abnormal recruitment of Syk to the T-cell receptor (TCR) complex. In these T-cells, FcεRIγ functionally substitutes for TCRζ and recruits Syk in place of ZAP70, which promotes T-cell activation and Syk phosphorylation that can be blocked by R406 [48]. Syk overexpression in normal T-cells or Syk knockdown in T-cells from lupus patients results in an exacerbation or normalization, respectively, of disease-related gene signatures [49]. Finally, Syk recruitment to phosphorylated ITAM-motifs in the adapter protein DAP12 is required for osteoclast function and so inhibitors of Syk may provide a novel therapeutic approach to diseases such as osteoporosis [50]. Therefore, altered Syk expression, recruitment, or activation may confer upon these disease states a dependence on Syk and an increased sensitivity to Syk inhibitors that may not otherwise exist.

CC-509 was the result of an optimization campaign toward improved selectivity with respect to the overall kinome and away from specific kinase targets such as KDR and Jak that likely contributed to aspects of the fostamatinib side-effect profile. A key liability of R406 which may underly some of the general tolerability issues seen in the clinic is promiscuity across the kinome. As shown here (S1 and S2 Tables), CC-509 is more selective than R406 across the kinome. In terms of KDR inhibition, we show that R406 inhibits cellular KDR activity at levels that are readily achievable in plasma from rodents and in human subjects [51]. Indeed, R406 induced hypertension, inhibited VEGF-induced hypotension, and blocked KDR phosphorylation *in vivo* in preclinical models [52], providing indirect but compelling evidence that hypertensive effect of R406 in the clinic may be mediated through KDR. In contrast, CC-509 inhibits KDR cell activity to comparable levels at 10-fold higher concentrations than R406 and at levels reached only briefly at minimum efficacious exposures in rodents (data not shown), suggesting it would be unlikely to trigger KDR-dependent hypertension in human patients. With respect to inhibition of Jak, CC-509 is less potent than R406 in the Jak2 enzyme (22 fold, data not shown), cellular (1.7 fold), and myeloid *in vitro* colony forming assays (3.1 to 5-fold). This fold difference-multiple is consistent with results obtained in preclinical toxicology studies with CC-509 in which the exposures producing myelosuppression, although not peripheral B-cell depletion (data not shown), in male rats is approximately 5-fold higher with CC-509 (630 μM·hr) than with R406 (120 μM·hr) [53]. At this high exposure CC-509 is predicted to cover Jak2 for 20 hours, while at the efficacious exposures (45 μM·hr) Jak2 is covered for 4 hours. Since experimental evidence suggests neutropenia is generally not observed using potent Jak inhibitors in rodents or human patients at doses in which Jak2 is inhibited for less than 8 hours [43,54, 55], CC-509 will be unlikely to induce neutropenia in human patients at exposures predicted to be efficacious. Based on the data presented here, we hypothesize that CC-509 will have a distinct and likely improved side effect profile in human patients when compared to fostamatinib. However, pre-clinical and clinical results will be required to conclusively demonstrate the potential safety advantages of CC-509.

Targeted Jak inhibitors such as baricitinib or tofacitinib have demonstrated activity in RA models and clinical trials [56]. It is unclear whether the efficacy of Syk inhibitors such as fostamatinib with activity against Jak is due to inhibition of Syk, Jak, or the combination. This has made interpretation of efficacy data difficult and has raised questions whether Syk can be considered a clinically validated target for RA. Recent preclinical evidence indicate selective Syk inhibitors that have no activity against Jak2 can be efficacious in models of arthritis [17,45]. In addition, a close analogue of CC-509 with negligible Jak2 activity is also efficacious in the CIA model (our unpublished results). These results suggest efficacy in models of arthritis can be achieved with Syk inhibition alone and does not require concomitant activity against Jak. In contrast to RA, it is very likely that efficacy observed in ITP by fostamatinib is driven primarily by Syk since Jak inhibitors cause thrombocytopenia and would be predicted to worsen the condition by blocking platelet survival signals [57].

Although fostamatinib ultimately did not succeed in the competitive RA clinical space, targeting Syk was a novel concept that has served to catalyze alternative approaches for treating RA and other autoimmune conditions. Rigel continues to develop fostamatinib in immune thrombocytopenia purpura (ITP) based on a Phase II trial involving patients with chronic and refractory ITP in which fostamatinib promoted durable platelet responses in 8/16 patients [58,59]. Other Syk kinase inhibitors have also had mixed results in clinical trials. The selective Syk inhibitor BIIB057 (PRT-2607, Portola Pharmaceuticals) exhibited selectivity across the kinome, potency in Syk-dependent cellular models and efficacy in models of arthritis [17]. BIIB057, although well tolerated in Phase I studies, was withdrawn from an RA Phase II trial for unknown reasons. A topically administered Syk/ Jak dual kinase inhibitor R333 (Rigel Pharmaceuticals) has completed Phase II trials in lupus erythematosus, but did not meet its primary endpoint [60]. HMPL-523 (Hutchinson Medi Pharma) is entering Phase I in healthy volunteers. Syk kinase inhibitors such as GS-9973 (Gilead Sciences) and PRT-2070 (Portola Pharmaceuticals) are positioned in the clinic for oncology indications. An extensive literature supporting a role for Syk and the use of Syk inhibitors in oncology exists [61], but is beyond the scope of this article.

In conclusion, we describe a novel triazolopyridine-based Syk kinase inhibitor, CC-509, with potent cellular activity in FcR-dependent systems, favorable PK properties, and efficacy in models of inflammatory disease and arthritis. Our studies with CC-509 support for the role of Syk in immunoreceptor-mediated signaling and confirm the efficacy of Syk inhibitors in models of inflammatory disease. Importantly, CC-509 was successfully optimized to improve overall kinase selectivity and activity against KDR and Jak, and has a clearly differentiated profile when compared to R406. Since none of the Syk compounds being developed are currently being positioned for RA, additional Syk candidates and clinical programs will be required to definitively demonstrate Syk as a clinically validated target. Based on its promising drug-like profile, CC-509 was nominated for clinical development.

## Supporting Information

S1 Table Kinase selectivity panel for CC-509. (DOCX)

S2 Table Chemical Structure and Kinase Selectivity Panel for R406. (DOCX)

## Abbreviations

Syk: spleen tyrosine kinase
FcR: immunoglobulin Fc-domain receptor
VEGFR2: vascular endothelial growth factor 2
KDR: kinase insert domain receptor
Jak: janus kinase
CIA: collagen induced arthritis
PCA: passive cutaneous anaphylaxis
RA: rheumatoid arthritis
q.d.: quaque die, once daily
b.i.d.: bis in die, twice daily
AUC: area-under-curve
IC50: half-maximal inhibitory concentration
Ki: inhibitory constant
TNF: tumor necrosis factor
NFAT: nuclear factor of activated T-cells
BCR: B-cell receptor
Hek: human embryonic kidney
TCR: T-cell receptor

## References

[1] Egerer K, Feist E, Burmester GR (2009) The serological diagnosis of rheumatoid arthritis: antibodies to citrullinated antigens. Deutsches Arzteblatt International 106:159–163. 10.3238/arztebl.2009.0159 19578391 PMC2695367

[2] Steiner G (2007) Auto-antibodies and autoreactive T-cells in rheumatoid arthritis: pathogenetic players and diagnostic tools. Clinical Reviews in Allergy & Immunology 32:23–36. 10.1007/BF02686079 17426358

[3] Morgan AW, Barrett JH, Griffiths B, Subramanian D, Robinson JI, Keyte VH, et al (2006) Analysis of Fcgamma receptor haplotypes in rheumatoid arthritis: FCGR3A remains a major susceptibility gene at this locus, with an additional contribution from FCGR3B. Arthritis Research & Therapy 8:R5. 16356189 10.1186/ar1847 PMC1526569

[4] Nieto A, Caliz R, Pascual M, Mataran L, Garcia S, Martin J (2000) Involvement of Fcgamma receptor IIIA genotypes in susceptibility to rheumatoid arthritis. Arthritis and Rheumatism 43:735–739. 10765917 10.1002/1529-0131(200004)43:4<735::AID-ANR3>3.0.CO;2-Q

[5] Firestein GS (2003) Evolving concepts of rheumatoid arthritis. Nature 423:356–361. 12748655 10.1038/nature01661

[6] Browning JL (2006) B cells move to centre stage: novel opportunities for autoimmune disease treatment. Nature Reviews Drug Discovery 5:564–576. 16816838 10.1038/nrd2085

[7] Wong BR, Grossbard EB, Payan DG, Masuda ES (2004) Targeting Syk as a treatment for allergic and autoimmune disorders. Expert Opinion on Investigational Drugs 13:743–762. 15212616 10.1517/13543784.13.7.743

[8] Kiefer F, Brumell J, Al-Alawi N, Latour S, Cheng A, Veillette A, et al (1998) The Syk protein tyrosine kinase is essential for Fcgamma receptor signaling in macrophages and neutrophils. Mol Cell Biol 18:4209–4220. 9632805 10.1128/mcb.18.7.4209 PMC109005

[9] Kurosaki T, Johnson SA, Pao L, Sada K, Yamamura H, Cambier JC (1995) Role of the Syk autophosphorylation site and SH2 domains in B cell antigen receptor signaling. J Exp Med 182:1815–1823. 7500027 10.1084/jem.182.6.1815 PMC2192262

[10] Costello PS, Turner M, Walters AE, Cunningham CN, Bauer PH, Downward J, et al (1996) Critical role for the tyrosine kinase Syk in signalling through the high affinity IgE receptor of mast cells. Oncogene 13:2595–2605. 9000133

[11] Cheng AM, Rowley B, Pao W, Hayday A, Bolen JB, Pawson T (1995) Syk tyrosine kinase required for mouse viability and B-cell development. Nature 378:303–306. 7477353 10.1038/378303a0

[12] Ozaki N, Suzuki S, Ishida M, Harada Y, Tanaka K, Sato Y, et al (2012) Syk-dependent signaling pathways in neutrophils and macrophages are indispensable in the pathogenesis of anti-collagen antibody-induced arthritis. International Immunology 24:539–550. 10.1093/intimm/dxs078 22914861

[13] Jakus Z, Simon E, Balazs B, Mocsai A. (2010) Genetic deficiency of Syk protects mice from autoantibody-induced arthritis. Arthritis Rheum. 62(7):1899–910. 10.1002/art.27438 20201079 PMC2972644

[14] Elliott ER, Van Ziffle JA, Scapini P, Sullivan BM, Locksley RM, Lowell CA (2010) Deletion of Syk in neutrophils prevents immune complex arthritis. J Immunol.187(8):4319–30. 10.4049/jimmunol.1100341 Epub 2011 Sep 14. PMC3186826 21918195

[15] Braselmann S, Taylor V, Zhao H, Wang S, Sylvain C, Baluom M, et al (2006) R406, an orally available spleen tyrosine kinase inhibitor blocks fc receptor signaling and reduces immune complex-mediated inflammation. J Pharmacol Exp Ther 319:998–1008. 16946104 10.1124/jpet.106.109058

[16] Pine PR, Chang B, Schoettler N, Banquerigo ML, Wang S, Lau A, et al (2007) Inflammation and bone erosion are suppressed in models of rheumatoid arthritis following treatment with a novel Syk inhibitor. Clinical Immunology 124:244–257. 17537677 10.1016/j.clim.2007.03.543

[17] Coffey G, DeGuzman F, Inagaki M, Pak Y, Delaney SM, Ives D, et al (2012) Specific inhibition of spleen tyrosine kinase suppresses leukocyte immune function and inflammation in animal models of rheumatoid arthritis. J Pharmacol Exp Ther 340:350–359. 10.1124/jpet.111.188441 22040680

[18] Singh R, Masuda ES, Payan DG (2012) Discovery and development of spleen tyrosine kinase (SYK) inhibitors. Journal of Medicinal Chemistry 55:3614–3643. 10.1021/jm201271b 22257213

[19] AstraZeneca (2013) AstraZeneca announces top-line results from Phase III OSKIRA Trials of FOSTAMATINIB and decision not to proceed with regulatory filings. http://www.astrazeneca.com: 06-04-2013

[20] National Institutes of Health (2014) BIIB057 in Subjects With Rheumatoid Arthritis and Inadequate Response to Disease-Modifying Antirheumatic Drugs (EMBRACE). http://www.clinicaltrials.gov: 11-01-2014

[21] Melnick JS, Janes J, Kim S, Chang JY, Sipes DG, Gunderson D, et al (2006) An efficient rapid system for profiling the cellular activities of molecular libraries. Proceedings of the National Academy of Sciences of the United States of America 103:3153–3158. 16492761 10.1073/pnas.0511292103 PMC1413928

[22] Kirshenbaum AS, Akin C, Wu Y, Rottem M, Goff JP, Beaven MA, et al (2003) Characterization of novel stem cell factor responsive human mast cell lines LAD 1 and 2 established from a patient with mast cell sarcoma/leukemia; activation following aggregation of FcepsilonRI or FcgammaRI. Leukemia Research 27:677–682. 12801524 10.1016/s0145-2126(02)00343-0

[23] Miyazawa K, Mori A, Okudaira H (1998) Establishment and characterization of a novel human rheumatoid fibroblast-like synoviocyte line, MH7A, immortalized with SV40 T antigen. Journal of Biochemistry 124:1153–1162. 9832620 10.1093/oxfordjournals.jbchem.a022233

[24] Otwinowski Z, Borek D, Majewski W, Minor W (2003) Multiparametric scaling of diffraction intensities. Acta crystallographica Section A, Foundations of Crystallography 59:228–234. 12714773 10.1107/s0108767303005488

[25] Murshudov GN, Vagin AA, Dodson EJ (1997) Refinement of macromolecular structures by the maximum-likelihood method. Acta crystallographica Section D, Biological Crystallography 53:240–255. 15299926 10.1107/S0907444996012255

[26] Trubetskoy OV, Gibson JR, Marks BD (2005) Highly miniaturized formats for in vitro drug metabolism assays using vivid fluorescent substrates and recombinant human cytochrome P450 enzymes. Journal of Biomolecular Screening 10:56–66. 15695344 10.1177/1087057104269731

[27] Loken MR, Brosnan JM, Bach BA, Ault KA (1990) Establishing optimal lymphocyte gates for immunophenotyping by flow cytometry. Cytometry 11:453–459. 1693112 10.1002/cyto.990110402

[28] Graczyk PP. (2007) Gini coefficient: a new way to express selectivity of kinase inhibitors against a family of kinases. J Med Chem 50(23):5773–9. Epub 2007 Oct 19 17948979 10.1021/jm070562u

[29] Kuno Y, Abe A, Emi N, Iida M, Yokozawa T, Towatari M, et al (2001) Constitutive kinase activation of the TEL-Syk fusion gene in myelodysplastic syndrome with t(9;12)(q22;p12). Blood 97:1050–1055. 11159536 10.1182/blood.v97.4.1050

[30] Hisamichi H, Naito R, Toyoshima A, Kawano N, Ichikawa A, Orita A, et al (2005) Synthetic studies on novel Syk inhibitors. Part 1: Synthesis and structure-activity relationships of pyrimidine-5-carboxamide derivatives. Bioorg & Med Chem 13:4936–4951. 15990316 10.1016/j.bmc.2005.05.033

[31] Lauzurica P, Sancho D, Torres M, Albella B, Marazuela M, Merino T, et al (2000) Phenotypic and functional characteristics of hematopoietic cell lineages in CD69-deficient mice. Blood 95:2312–2320. 10733501

[32] Eberlein-Konig B, Rakoski J, Behrendt H, Ring J (2004) Use of CD63 expression as marker of in vitro basophil activation in identifying the culprit in insect venom allergy. Journal of Investigational Allergology & Clinical Immunology 14:10–16. 15160437

[33] Keystone EC, Schorlemmer HU, Pope C, Allison AC (1977) Zymosan-induced arthritis: a model of chronic proliferative arthritis following activation of the alternative pathway of complement. Arthritis and Rheumatism 20:1396–1401. 911357 10.1002/art.1780200714

[34] Forsberg M, Lofgren R, Zheng L, Stendahl O (2001) Tumour necrosis factor-alpha potentiates CR3-induced respiratory burst by activating p38 MAP kinase in human neutrophils. Immunology 103:465–472. 11529937 10.1046/j.1365-2567.2001.01270.x PMC1783267

[35] Underhill DM, Rossnagle E, Lowell CA, Simmons RM (2005) Dectin-1 activates Syk tyrosine kinase in a dynamic subset of macrophages for reactive oxygen production. Blood. 106(7):2543–50. Epub 2005 Jun 14. 15956283 10.1182/blood-2005-03-1239 PMC1895265

[36] Tsubokawa M, Tohyama Y, Tohyama K, Asahi M, Inazu T, Nakamura H, et al (1997) Interleukin-3 activates Syk in a human myeloblastic leukemia cell line, AML193. European Journal of Biochemistry / FEBS 249(3):792–6. 9395328 10.1111/j.1432-1033.1997.t01-2-00792.x

[37] Cha HS, Boyle DL, Inoue T, Schoot R, Tak PP, Pine P, et al (2006) A novel spleen tyrosine kinase inhibitor blocks c-Jun N-terminal kinase-mediated gene expression in synoviocytes. Journal of Pharmacology and Experimental Therapeutics 317:571–578. 16452391 10.1124/jpet.105.097436

[38] Bartok B, Firestein GS (2010) Fibroblast-like synoviocytes: key effector cells in rheumatoid arthritis. Immunological Reviews 233:233–255. 10.1111/j.0105-2896.2009.00859.x 20193003 PMC2913689

[39] Nijjar JS, Tindell A, McInnes IB, Siebert S (2013) Inhibition of spleen tyrosine kinase in the treatment of rheumatoid arthritis. Rheumatology 52:1556–1562. 10.1093/rheumatology/ket225 23861534

[40] Salgado E, Maneiro JR, Carmona L, Gomez-Reino JJ (2014) Safety profile of protein kinase inhibitors in rheumatoid arthritis: systematic review and meta-analysis. Annals of the Rheumatic Diseases 73:871–882. 10.1136/annrheumdis-2012-203116 23599436

[41] Davis MI, Hunt JP, Herrgard S, Ciceri P, Wodicka LM, Pallares G, et al (2011) Comprehensive analysis of kinase inhibitor selectivity. Nature Biotechnology 29:1046–1051. 10.1038/nbt.1990 22037378

[42] Grandage VL, Everington T, Linch DC, Khwaja A (2006) Go6976 is a potent inhibitor of the JAK 2 and FLT3 tyrosine kinases with significant activity in primary acute myeloid leukaemia cells. British Journal of Haematology 135:303–316. 16956345 10.1111/j.1365-2141.2006.06291.x

[43] Meyer DM, Jesson MI, Li X, Elrick MM, Funckes-Shippy CL, Warner JD, et al (2010) Anti-inflammatory activity and neutrophil reductions mediated by the JAK1/JAK3 inhibitor, CP-690,550, in rat adjuvant-induced arthritis. Journal of Inflammation 7:41–53. 10.1186/1476-9255-7-41 20701804 PMC2928212

[44] Ravetch JV, Bolland S (2001) IgG Fc receptors. Annual Review of Immunology 19:275–290. 11244038 10.1146/annurev.immunol.19.1.275

[45] Liao C, Hsu J, Kim Y, Hu DQ, Xu D, Zhang J, et al (2013) Selective inhibition of spleen tyrosine kinase (SYK) with a novel orally bioavailable small molecule inhibitor, RO9021, impinges on various innate and adaptive immune responses: implications for SYK inhibitors in autoimmune disease therapy. Arthritis Research & Therapy 15:R146. 24286216 10.1186/ar4329 PMC3978604

[46] Holmdahl R, Mo J, Nordling C, Larsson P, Jansson L, Goldschmidt T, et al (1989) Collagen induced arthritis: an experimental model for rheumatoid arthritis with involvement of both DTH and immune complex mediated mechanisms. Clinical and Experimental Rheumatology Suppl 3:S51–S55. 2691160

[47] Brenner M, Gulko PS (2012) The arthritis severity locus Cia5a regulates the expression of inflammatory mediators including Syk pathway genes and proteases in pristane-induced arthritis. BMC Genomics 13:710 10.1186/1471-2164-13-710 23249408 PMC3548698

[48] Krishnan S, Juang YT, Chowdhury B, Magilavy A, Fisher CU, Nguyen H, et al (2008) Differential expression and molecular associations of Syk in systemic lupus erythematosus T cells. Journal of Immunology 181:8145–8152. 10.4049/jimmunol.181.11.8145 PMC2586973 19018007

[49] Grammatikos AP, Ghosh D, Devlin A, Kyttaris VC, Tsokos GC (2013) Spleen tyrosine kinase (Syk) regulates systemic lupus erythematosus (SLE) T cell signaling. PLOS One 8:e74550 10.1371/journal.pone.0074550 24013589 PMC3754955

[50] Mocsai A, Humphrey MB, Van Ziffle JA, Hu Y, Burghardt A, Spusta SC, et al (2004) The immunomodulatory adapter proteins DAP12 and Fc receptor gamma-chain (FcRgamma) regulate development of functional osteoclasts through the Syk tyrosine kinase. Proc Natl Acad Sci U S A. 101(16):6158–63. Epub 2004 Apr 8 15073337 10.1073/pnas.0401602101 PMC395939

[51] Baluom M, Grossbard EB, Mant T, Lau DT (2013) Pharmacokinetics of fostamatinib, a spleen tyrosine kinase (SYK) inhibitor, in healthy human subjects following single and multiple oral dosing in three phase I studies. British Journal of Clinical Pharmacology 76:78–88. 10.1111/bcp.12048 PMC3703230 23190017

[52] Skinner M, Philp K, Lengel D, Coverley L, Lamm Bergstrom E, Glaves P, et al (2014) The Contribution of Vegf Signalling to Fostamatinib-Induced Blood Pressure Elevation. British Journal of Pharmacology 171:2308–20 10.1111/bph.12559 24329544 PMC3997272

[53] Zhu Y, Herlaar E, Masuda ES, Burleson GR, Nelson AJ, Grossbard EB, et al (2007) Immunotoxicity assessment for the novel Spleen tyrosine kinase inhibitor R406. Toxicology and Applied Pharmacology 221:268–277. 17490694 10.1016/j.taap.2007.03.027

[54] Fridman JS, Scherle PA, Collins R, Burn TC, Li Y, Li J, et al (2010) Selective inhibition of JAK1 and JAK2 is efficacious in rodent models of arthritis: preclinical characterization of INCB028050. Journal of Immunology 184:5298–5307. 10.4049/jimmunol.0902819 20363976

[55] Quintas-Cardama A, Vaddi K, Liu P, Manshouri T, Li J, Scherle PA, et al (2010) Preclinical characterization of the selective JAK1/2 inhibitor INCB018424: therapeutic implications for the treatment of myeloproliferative neoplasms. Blood 115:3109–3117. 10.1182/blood-2009-04-214957 20130243 PMC3953826

[56] Gadina M (2013) Janus kinases: an ideal target for the treatment of autoimmune diseases. Journal of Investigative Dermatology Symposium Proceedings 16:S70–S72. 10.1038/jidsymp.2013.29 PMC4181323 24326567

[57] Geddis AE, Linden HM, Kaushansky K (2002) Thrombopoietin: a pan-hematopoietic cytokine. Cytokine & Growth Factor Reviews 13:61–73. 10.1016/s1359-6101(01)00030-2 11750880

[58] Podolanczuk A, Lazarus AH, Crow AR, Grossbard E, Bussel JB (2009) Of mice and men: an open-label pilot study for treatment of immune thrombocytopenic purpura by an inhibitor of Syk. Blood 113:3154–3160. 10.1182/blood-2008-07-166439 19096013

[59] Rigel Pharmaceuticals (2013) Rigel Provides Pipeline Update. http://ir.rigel.com: 10-24-2013.

[60] Genetic Engineering and Biotechnology News. Rigel Scuttles R333 after Phase II Flop. http://www.genengnews.com/gen-news-highlights/rigel-scuttles-r333-after-phase-ii-flop/81249024: 10-25-2013.

[61] Efremov DG, Laurenti L (2011) The Syk kinase as a therapeutic target in leukemia and lymphoma. Expert Opinion on Investigational Drugs 20:623–636. 10.1517/13543784.2011.570329 21438742

