# Supplementary material for: A Novel Triazolopyridine-Based Spleen Tyrosine Kinase Inhibitor That Arrests Joint Inflammation
Source: PLoS One. 2016 Jan 12;11(1):e0145705. doi: 10.1371/journal.pone.0145705 (PMC4710522; doi:10.1371/journal.pone.0145705)
Supplement: S1 Table — (DOCX) [file pone.0145705.s001.docx]

**S1 Table. Kinase selectivity panel for CC-509.**

Kinase activity was measured using either the Z’-Lyte^®^ or the Adapta^®^ protocols as part of the SelectScreen^™^ profiling service offered at Life Technologies (Grand Island, NY). For the single point measurements (Part A), CC-509 was kept constant at 3 µM while ATP concentrations [ATP] used were at the K_M_ for ATP (KM ATP) for that specific enyzme, at 10 or 100 µM ATP (10 ATP or 100 ATP, respectively), as indicated. Values expressed as percent control (% control) relative to DMSO-only wells (uninhibited, 100%). For full dose-response-curves (Part B), CC-509 was titrated in 10-point, 3-fold serial dilutions starting at 30 µM and IC50 values were calculated based on percent control values using standard curve fitting methods.

| Kinase (single point: 3 µM CC-509) | [ATP] | % control |
| --- | --- | --- |
| Invitrogen_1pt Abl_E255K_h | KM ATP | 26 |
| Invitrogen_1pt Abl_G250E_h | KM ATP | 29 |
| Invitrogen_1pt Abl_h | KM ATP | 19 |
| Invitrogen_1pt Abl_T315I_h | KM ATP | 34 |
| Invitrogen_1pt Abl_Y253F_h | KM ATP | 22 |
| Invitrogen_1pt ALK_h | KM ATP | 45 |
| Invitrogen_1pt ALK4_h | KM ATP | 107 |
| Invitrogen_1pt AMPK_A2_B1_G1_h | KM ATP | 63 |
| Invitrogen_1pt AMPK_r | KM ATP | 84 |
| Invitrogen_1pt Arg_m | KM ATP | 31 |
| Invitrogen_1pt ARK5_h | KM ATP | 7 |
| Invitrogen_1pt Aurora-A_h | KM ATP | 6 |
| Invitrogen_1pt Aurora-B_h | KM ATP | 14 |
| Invitrogen_1pt Aurora-C_h | KM ATP | 18 |
| Invitrogen_1pt Axl_h | KM ATP | 70 |
| Invitrogen_1pt Blk_m | KM ATP | 63 |
| Invitrogen_1pt Bmx_h | KM ATP | 52 |
| Invitrogen_1pt BRAF | 100 ATP | 83 |
| Invitrogen_1pt BRAF V599E | 100 ATP | 88 |
| Invitrogen_1pt BRK_h | KM ATP | 87 |
| Invitrogen_1pt BrSK1_h | KM ATP | 65 |
| Invitrogen_1pt BTK_h | KM ATP | 72 |
| Invitrogen_1pt CAMK1_h | 10 ATP | 86 |
| Invitrogen_1pt CaMKI-delta_h | KM ATP | 86 |
| Invitrogen_1pt CaMKII-alpha_h | KM ATP | 80 |
| Invitrogen_1pt CaMKII-beta_h | KM ATP | 104 |
| Invitrogen_1pt CaMKIV_h | KM ATP | 100 |
| Invitrogen_1pt CDK1_cyclinB_h | KM ATP | 82 |
| Invitrogen_1pt CDK2_cyclinA_h | KM ATP | 64 |
| Invitrogen_1pt CDK5_p25_h | KM ATP | 82 |
| Invitrogen_1pt CDK5_p35_h | KM ATP | 77 |
| Invitrogen_1pt CDK7_h | KM ATP | 94 |
| Invitrogen_1pt CDK9_h | KM ATP | 31 |
| Invitrogen_1pt CHK1_h | KM ATP | 94 |
| Invitrogen_1pt CHK2_h | KM ATP | 97 |
| Invitrogen_1pt CK1-alpha1_h | KM ATP | 88 |
| Invitrogen_1pt CK1-delta_h | KM ATP | 88 |
| Invitrogen_1pt CK1-epsilon_h | KM ATP | 89 |
| Invitrogen_1pt CK1-gamma1_h | KM ATP | 54 |
| Invitrogen_1pt CK1-gamma2_h | KM ATP | 68 |
| Invitrogen_1pt CK1-gamma3_h | KM ATP | 50 |
| Invitrogen_1pt CK2_h | KM ATP | 94 |
| Invitrogen_1pt CK2-alpha2_h | KM ATP | 97 |
| Invitrogen_1pt cKit_h | KM ATP | 66 |
| Invitrogen_1pt cKit_T670I_h | KM ATP | 83 |
| Invitrogen_1pt CLK1_h | KM ATP | 82 |
| Invitrogen_1pt CLK2_h | KM ATP | 50 |
| Invitrogen_1pt CLK3_h | KM ATP | 81 |
| Invitrogen_1pt c-RAF_h | 100 ATP | 70 |
| Invitrogen_1pt CSK_h | KM ATP | 70 |
| Invitrogen_1pt cSRC_h | KM ATP | 67 |
| Invitrogen_1pt cSRC-N1_h | KM ATP | 58 |
| Invitrogen_1pt DAPK1_h | KM ATP | 104 |
| Invitrogen_1pt DCAMKL2_h | KM ATP | 98 |
| Invitrogen_1pt DYR1A_h | KM ATP | 99 |
| Invitrogen_1pt DYRK1B_h | KM ATP | 95 |
| Invitrogen_1pt DYRK3_h | KM ATP | 80 |
| Invitrogen_1pt DYRK4_h | KM ATP | 97 |
| Invitrogen_1pt EEF2K_h | KM ATP | 94 |
| Invitrogen_1pt EGFR_h | KM ATP | 97 |
| Invitrogen_1pt EGFR_L858R_h | KM ATP | 101 |
| Invitrogen_1pt EGFR_L861Q_h | KM ATP | 97 |
| Invitrogen_1pt EGFR_T790M_h | KM ATP | 54 |
| Invitrogen_1pt EGFR_T790M_L858R_h | KM ATP | 52 |
| Invitrogen_1pt EphA1_h | KM ATP | 21 |
| Invitrogen_1pt EphA2_h | KM ATP | 82 |
| Invitrogen_1pt EphA3_h | KM ATP | 98 |
| Invitrogen_1pt EphA4_h | KM ATP | 76 |
| Invitrogen_1pt EphA5_h | KM ATP | 89 |
| Invitrogen_1pt EphA8_h | KM ATP | 91 |
| Invitrogen_1pt EphB1_h | KM ATP | 41 |
| Invitrogen_1pt EphB2_h | KM ATP | 75 |
| Invitrogen_1pt EphB3_h | KM ATP | 42 |
| Invitrogen_1pt EphB4_h | KM ATP | 87 |
| Invitrogen_1pt ErbB4_h | KM ATP | 91 |
| Invitrogen_1pt FAK_h | KM ATP | 43 |
| Invitrogen_1pt Fer_h | KM ATP | 40 |
| Invitrogen_1pt Fes_h | KM ATP | 53 |
| Invitrogen_1pt FGFR1_h | KM ATP | 69 |
| Invitrogen_1pt FGFR2_h | KM ATP | 46 |
| Invitrogen_1pt FGFR3_h | KM ATP | 68 |
| Invitrogen_1pt FGFR3_K650E_h | KM ATP | 54 |
| Invitrogen_1pt FGFR4_h | KM ATP | 68 |
| Invitrogen_1pt Fgr_h | KM ATP | 21 |
| Invitrogen_1pt Flt1_h | KM ATP | 55 |
| Invitrogen_1pt Flt3_D835Y_h | KM ATP | -1 |
| Invitrogen_1pt Flt3_h | KM ATP | 6 |
| Invitrogen_1pt Flt4_h | KM ATP | 1 |
| Invitrogen_1pt Fms_h | KM ATP | 2 |
| Invitrogen_1pt Fyn_h | KM ATP | 74 |
| Invitrogen_1pt Gck_h | KM ATP | 57 |
| Invitrogen_1pt GRK2_h | KM ATP | 82 |
| Invitrogen_1pt GRK3_h | KM ATP | 98 |
| Invitrogen_1pt GRK4_h | KM ATP | 82 |
| Invitrogen_1pt GRK5_h | KM ATP | 96 |
| Invitrogen_1pt GRK6_h | KM ATP | 92 |
| Invitrogen_1pt GRK7_h | KM ATP | 94 |
| Invitrogen_1pt GSG2 _h | KM ATP | 100 |
| Invitrogen_1pt GSK3-alpha_h | KM ATP | 91 |
| Invitrogen_1pt GSK3-beta_h | KM ATP | 88 |
| Invitrogen_1pt Hck_h | KM ATP | 71 |
| Invitrogen_1pt Hgk_h | KM ATP | 62 |
| Invitrogen_1pt HIPK1_h | KM ATP | 94 |
| Invitrogen_1pt HIPK2_h | KM ATP | 96 |
| Invitrogen_1pt HIPK4_h | KM ATP | 100 |
| Invitrogen_1pt IGF-1R_h | KM ATP | 97 |
| Invitrogen_1pt IKBKE_h | KM ATP | 67 |
| Invitrogen_1pt IKK1_h | KM ATP | 106 |
| Invitrogen_1pt IKK-beta_h | KM ATP | 99 |
| Invitrogen_1pt IR_h | KM ATP | 91 |
| Invitrogen_1pt IRAK1_h | KM ATP | 20 |
| Invitrogen_1pt IRAK4_h | KM ATP | 94 |
| Invitrogen_1pt IRR_h | KM ATP | 99 |
| Invitrogen_1pt Itk_h | KM ATP | 91 |
| Invitrogen_1pt JAK1_h | KM ATP | 0 |
| Invitrogen_1pt JAK2_h | KM ATP | 4 |
| Invitrogen_1pt JAK2-JH1-JH2_h | KM ATP | 4 |
| Invitrogen_1pt JAK2-JH1-JH2_V617F_h | KM ATP | 8 |
| Invitrogen_1pt JAK3_h | KM ATP | 5 |
| Invitrogen_1pt JNK1-alpha1_h | 100 ATP | 88 |
| Invitrogen_1pt JNK2-alpha2_h | 100 ATP | 88 |
| Invitrogen_1pt JNK3_h | 100 ATP | 94 |
| Invitrogen_1pt KDR_h | KM ATP | 3 |
| Invitrogen_1pt Khs_1 | KM ATP | 81 |
| Invitrogen_1pt Lck_h | KM ATP | 39 |
| Invitrogen_1pt LRRK2 G2019S_h | KM ATP | 4 |
| Invitrogen_1pt LRRK2_h | KM ATP | 3 |
| Invitrogen_1pt Ltk_h | KM ATP | 71 |
| Invitrogen_1pt LYN-A_h | KM ATP | 54 |
| Invitrogen_1pt LYN-B_h | KM ATP | 39 |
| Invitrogen_1pt MAP3K8_h | 100 ATP | 79 |
| Invitrogen_1pt MAPK1_h | KM ATP | 94 |
| Invitrogen_1pt MAPK3_h | KM ATP | 90 |
| Invitrogen_1pt MAPKAP-K2_h | KM ATP | 96 |
| Invitrogen_1pt MAPKAP-K3_h | KM ATP | 88 |
| Invitrogen_1pt MARK1_h | KM ATP | 48 |
| Invitrogen_1pt MARK2_h | KM ATP | 55 |
| Invitrogen_1pt MARK3_h | KM ATP | 42 |
| Invitrogen_1pt MARK4_h | KM ATP | 33 |
| Invitrogen_1pt MATK_h | KM ATP | 102 |
| Invitrogen_1pt MEK1_h | 100 ATP | 65 |
| Invitrogen_1pt MEK2_h | 100 ATP | 73 |
| Invitrogen_1pt MELK_h | KM ATP | 10 |
| Invitrogen_1pt Mer_h | KM ATP | 90 |
| Invitrogen_1pt Met_h | KM ATP | 66 |
| Invitrogen_1pt Met_M1250T_h | KM ATP | 74 |
| Invitrogen_1pt MINK_h | KM ATP | 55 |
| Invitrogen_1pt MKK6_h | 100 ATP | 101 |
| Invitrogen_1pt MLK1_h | KM ATP | 2 |
| Invitrogen_1pt MRCK-alpha_h | KM ATP | 91 |
| Invitrogen_1pt MRCK-beta_h | KM ATP | 94 |
| Invitrogen_1pt MSK1_h | KM ATP | 94 |
| Invitrogen_1pt MSK2_h | KM ATP | 81 |
| Invitrogen_1pt MSSK1_h | KM ATP | 98 |
| Invitrogen_1pt MST1_h | KM ATP | 65 |
| Invitrogen_1pt MST2_h | KM ATP | 46 |
| Invitrogen_1pt MST3_h | KM ATP | 107 |
| Invitrogen_1pt MST4_h | KM ATP | 99 |
| Invitrogen_1pt mTOR_h | KM ATP | 92 |
| Invitrogen_1pt MuSK_h | KM ATP | 41 |
| Invitrogen_1pt MYLK2_h | KM ATP | 80 |
| Invitrogen_1pt NEK1_h | KM ATP | 73 |
| Invitrogen_1pt NEK2_h | KM ATP | 101 |
| Invitrogen_1pt NEK4_h | KM ATP | 78 |
| Invitrogen_1pt NEK6_h | KM ATP | 99 |
| Invitrogen_1pt NEK7_h | KM ATP | 102 |
| Invitrogen_1pt NEK9_h | KM ATP | 95 |
| Invitrogen_1pt p38-alpha_h | 100 ATP | 89 |
| Invitrogen_1pt p38-beta_h | KM ATP | 84 |
| Invitrogen_1pt p38-delta_h | KM ATP | 82 |
| Invitrogen_1pt p38-gamma_h | KM ATP | 95 |
| Invitrogen_1pt p70S6K_h | KM ATP | 89 |
| Invitrogen_1pt PAK1_h | KM ATP | 94 |
| Invitrogen_1pt PAK2_h | KM ATP | 85 |
| Invitrogen_1pt PAK3_h | KM ATP | 97 |
| Invitrogen_1pt PAK4_h | KM ATP | 87 |
| Invitrogen_1pt PAK6_h | KM ATP | 91 |
| Invitrogen_1pt PAK7_h | KM ATP | 86 |
| Invitrogen_1pt PASK_h | KM ATP | 100 |
| Invitrogen_1pt PDGFR-alpha_D842V_h | KM ATP | 9 |
| Invitrogen_1pt PDGFR-alpha_h | KM ATP | 4 |
| Invitrogen_1pt PDGFR-alpha_T674I_h | KM ATP | 80 |
| Invitrogen_1pt PDGFR-alpha_V561D_h | KM ATP | 0 |
| Invitrogen_1pt PDGFR-beta_h | KM ATP | 43 |
| Invitrogen_1pt PDK1_h | KM ATP | 47 |
| Invitrogen_1pt PDK1_h | 100 ATP | 78 |
| Invitrogen_1pt PhK-gamma1_h | KM ATP | 75 |
| Invitrogen_1pt PhK-gamma2_h | KM ATP | 103 |
| Invitrogen_1pt PI3_Kinase-alpha_h | KM ATP | 81 |
| Invitrogen_1pt PI3_Kinase-delta_h | KM ATP | 58 |
| Invitrogen_1pt PI3_Kinase-gamma_h | KM ATP | 61 |
| Invitrogen_1pt PI3K-C2 alpha_h | KM ATP | 95 |
| Invitrogen_1pt PI3K-C2 beta_h | 10 ATP | 98 |
| Invitrogen_1pt PI4K-alpha_h | 10 ATP | 120 |
| Invitrogen_1pt PI4K-beta_h | KM ATP | 77 |
| Invitrogen_1pt PIK3C3_h | KM ATP | 79 |
| Invitrogen_1pt Pim-1_h | KM ATP | 101 |
| Invitrogen_1pt Pim-2_h | KM ATP | 99 |
| Invitrogen_1pt PKA_h | KM ATP | 93 |
| Invitrogen_1pt PKB-alpha_h | KM ATP | 100 |
| Invitrogen_1pt PKB-beta_h | KM ATP | 98 |
| Invitrogen_1pt PKB-gamma_h | KM ATP | 99 |
| Invitrogen_1pt PKC-alpha_h | KM ATP | 71 |
| Invitrogen_1pt PKC-betaI_h | KM ATP | 92 |
| Invitrogen_1pt PKC-betaII_h | KM ATP | 43 |
| Invitrogen_1pt PKC-delta_h | KM ATP | 91 |
| Invitrogen_1pt PKC-epsilon_h | KM ATP | 100 |
| Invitrogen_1pt PKC-eta_h | KM ATP | 102 |
| Invitrogen_1pt PKC-gamma_h | KM ATP | 45 |
| Invitrogen_1pt PKC-iota_h | KM ATP | 88 |
| Invitrogen_1pt PKC-mu_h | KM ATP | 34 |
| Invitrogen_1pt PKC-theta_h | KM ATP | 78 |
| Invitrogen_1pt PKC-zeta_h | KM ATP | 86 |
| Invitrogen_1pt PKD2_h | KM ATP | 52 |
| Invitrogen_1pt PKD3_h | KM ATP | 37 |
| Invitrogen_1pt Plk1_h | KM ATP | 88 |
| Invitrogen_1pt Plk2_h | KM ATP | 96 |
| Invitrogen_1pt Plk3_h | KM ATP | 88 |
| Invitrogen_1pt PRAK_h | KM ATP | 91 |
| Invitrogen_1pt PRK1_h | KM ATP | 60 |
| Invitrogen_1pt PRKG1_h | KM ATP | 96 |
| Invitrogen_1pt PRKG2_h | KM ATP | 93 |
| Invitrogen_1pt PrKX_h | KM ATP | 95 |
| Invitrogen_1pt PTK2B_h | KM ATP | 50 |
| Invitrogen_1pt PTK5_h | KM ATP | 86 |
| Invitrogen_1pt Ret_h | KM ATP | 13 |
| Invitrogen_1pt Ret_V804L_h | KM ATP | 22 |
| Invitrogen_1pt Ret_Y791F_h | KM ATP | 18 |
| Invitrogen_1pt ROCK-I_h | KM ATP | 70 |
| Invitrogen_1pt ROCK-II_h | KM ATP | 69 |
| Invitrogen_1pt Ron_h | KM ATP | 74 |
| Invitrogen_1pt Ros_h | KM ATP | 74 |
| Invitrogen_1pt Rse_h | KM ATP | 53 |
| Invitrogen_1pt Rsk1_h | KM ATP | 60 |
| Invitrogen_1pt Rsk2_h | KM ATP | 49 |
| Invitrogen_1pt Rsk3_h | KM ATP | 33 |
| Invitrogen_1pt Rsk4_h | KM ATP | 32 |
| Invitrogen_1pt SGK_h | KM ATP | 96 |
| Invitrogen_1pt SGK2_h | KM ATP | 89 |
| Invitrogen_1pt SGK3_h | KM ATP | 87 |
| Invitrogen_1pt SIK2_h | KM ATP | 6 |
| Invitrogen_1pt SPHK1_h | KM ATP | 96 |
| Invitrogen_1pt SPHK2_h | 10 ATP | 105 |
| Invitrogen_1pt SRMS_h | KM ATP | 90 |
| Invitrogen_1pt SRPK1_h | KM ATP | 98 |
| Invitrogen_1pt SRPK2_h | KM ATP | 111 |
| Invitrogen_1pt STK25_h | KM ATP | 97 |
| Invitrogen_1pt Syk_h | KM ATP | -2 |
| Invitrogen_1pt TAO1_h | KM ATP | 96 |
| Invitrogen_1pt TBK1_h | KM ATP | 10 |
| Invitrogen_1pt Tie2_h | KM ATP | 87 |
| Invitrogen_1pt TrkA_h | KM ATP | 41 |
| Invitrogen_1pt TrkB_h | KM ATP | 34 |
| Invitrogen_1pt TrkC_h | KM ATP | 25 |
| Invitrogen_1pt TSSK1_h | KM ATP | 62 |
| Invitrogen_1pt TSSK2_h | KM ATP | 93 |
| Invitrogen_1pt Tyk2_h | KM ATP | 3 |
| Invitrogen_1pt Yes_h | KM ATP | 54 |
| Invitrogen_1pt ZAP-70_h | KM ATP | 70 |
| Invitrogen_1pt ZIPK_h | KM ATP | 96 |
|  |  |  |
| Kinase (CC-509 dose-response) | IC50 value |  |
| Invitrogen_DRC Aurora-B_h | 0.4091 µM |  |
| Invitrogen_DRC cKit_h | 11.023 µM |  |
| Invitrogen_DRC Flt3_h | 0.14297 µM |  |
| Invitrogen_DRC Flt4_h | 0.053947 µM |  |
| Invitrogen_DRC Fms_h | 0.008612 µM |  |
| Invitrogen_DRC JAK1_h | 0.043987 µM |  |
| Invitrogen_DRC JAK2_h | 0.018946 µM |  |
| Invitrogen_DRC JAK3_h | 0.018041 µM |  |
| Invitrogen_DRC KDR_h | 0.18451 µM |  |
| Invitrogen_DRC LYN-A_h | 3.5811 µM |  |
| Invitrogen_DRC PDGFR-alpha_h | 0.20392 µM |  |
| Invitrogen_DRC Ret_h | 0.39666 µM |  |
| Invitrogen_DRC Syk_h | 0.027303 µM |  |
| Invitrogen_DRC Tyk2_h | 0.11241 µM |  |
| Invitrogen_DRC ZAP-70_h | >3 µM |  |
